# Subtle Roles of Down Syndrome Cell Adhesion Molecules in Embryonic Forebrain Development and Neuronal Migration

**DOI:** 10.3389/fcell.2020.624181

**Published:** 2021-01-28

**Authors:** Manuela D. Mitsogiannis, Anna Pancho, Tania Aerts, Sonja M. Sachse, Ria Vanlaer, Lut Noterdaeme, Dietmar Schmucker, Eve Seuntjens

**Affiliations:** ^1^Developmental Neurobiology Group, Animal Physiology and Neurobiology Division, Department of Biology, Katholieke Universiteit Leuven, Leuven, Belgium; ^2^Neuronal Wiring Laboratory, Department of Neurosciences, VIB-KU Leuven Center for Brain & Disease Research, Katholieke Universiteit Leuven, Leuven, Belgium; ^3^Neuronal Wiring Group, Life & Medical Sciences Institute, University of Bonn, Bonn, Germany

**Keywords:** Dscam, Dscaml1, neuronal migration, cell adhesion, telencephalic development, radial migration, interneuron migration

## Abstract

Down Syndrome (DS) Cell Adhesion Molecules (DSCAMs) are transmembrane proteins of the immunoglobulin superfamily. Human DSCAM is located within the DS critical region of chromosome 21 (duplicated in Down Syndrome patients), and mutations or copy-number variations of this gene have also been associated to Fragile X syndrome, intellectual disability, autism, and bipolar disorder. The DSCAM paralogue DSCAM-like 1 (DSCAML1) maps to chromosome 11q23, implicated in the development of Jacobsen and Tourette syndromes. Additionally, a spontaneous mouse DSCAM deletion leads to motor coordination defects and seizures. Previous research has revealed roles for DSCAMs in several neurodevelopmental processes, including synaptogenesis, dendritic self-avoidance, cell sorting, axon growth and branching. However, their functions in embryonic mammalian forebrain development have yet to be completely elucidated. In this study, we revealed highly dynamic spatiotemporal patterns of *Dscam* and *Dscaml1* expression in definite cortical layers of the embryonic mouse brain, as well as in structures and ganglionic eminence-derived neural populations within the embryonic subpallium. However, an in-depth histological analysis of cortical development, ventral forebrain morphogenesis, cortical interneuron migration, and cortical-subcortical connectivity formation processes in Dscam and Dscaml1 knockout mice (*Dscam*^*del*17^ and *Dscaml1*^*GT*^) at several embryonic stages indicated that constitutive loss of *Dscam* and *Dscaml1* does not affect these developmental events in a significant manner. Given that several *Dscam*- and *Dscaml1*-linked neurodevelopmental disorders are associated to chromosomal region duplication events, we furthermore sought to examine the neurodevelopmental effects of *Dscam* and *Dscaml1* gain of function (GOF). *In vitro, ex vivo*, and *in vivo* GOF negatively impacted neural migration processes important to cortical development, and affected the morphology of maturing neurons. Overall, these findings contribute to existing knowledge on the molecular etiology of human neurodevelopmental disorders by elucidating how dosage variations of genes encoding adhesive cues can disrupt cell-cell or cell-environment interactions crucial for neuronal migration.

## Introduction

Down Syndrome (DS) Cell Adhesion Molecules (DSCAMs) represent a small group of transmembrane proteins of the immunoglobulin superfamily comprising, in vertebrates, DSCAM and its paralogue DSCAM-like 1 (DSCAML1) (Yamakawa et al., 1998; Agarwala et al., 2001). These molecules owe their name to the location of human *DSCAM* within the DS critical region of chromosome 21 (Yamakawa et al., 1998; Schmucker and Chen, 2009), which is considered to be crucially involved in the emergence of cognitive phenotypes associated with DS (Delabar et al., 1993; Korenberg et al., 1994; Belichenko et al., 2009, 2015; Aziz et al., 2018). Higher DSCAM levels have been observed in post-mortem brain tissue preparations/cultures from DS-affected patients and fetuses (Saito et al., 2000; Bahn et al., 2002), as well as in the central nervous system (CNS) of DS mouse models (Alves-Sampaio et al., 2010).

In addition to trisomy 21, mutations, single-nucleotide polymorphisms (SNPs), and transcriptional dysregulation of this gene have also been associated to other neurodevelopmental and neuropsychiatric disorders, including Fragile X syndrome (Brown et al., 2001; Darnell et al., 2011; Ascano et al., 2012; Cvetkovska et al., 2013), intellectual disability (Wei et al., 2016; Aleksiuniene et al., 2017; Monies et al., 2017; Stessman et al., 2017), autism (Iossifov et al., 2014; Turner et al., 2016; Wang et al., 2016; Varghese et al., 2017), bipolar disorder (Amano et al., 2008), and epilepsy (Shen et al., 2011; Wei et al., 2016). Animal models further substantiate a causal relation between variations in *Dscam* gene dosage and CNS dysfunction. A spontaneous *Dscam* null mutation occurring in mice (*Dscam*^*del*17^) leads to the early post-natal emergence of uncoordinated movements; as adults, these animals additionally display severe hydrocephalus, seizures, aberrant locomotion, and impaired motor learning (Fuerst et al., 2008; Xu et al., 2011). Similarly, mice carrying a different *Dscam* null mutant allele (*Dscam*^2*J*^) present dystonic hypertonia and deficits in locomotor coordination related to abnormalities in central sensorimotor circuitry (Fuerst et al., 2010; Lemieux et al., 2016; Thiry et al., 2016, 2018; Laflamme et al., 2019). Viability of *Dscam* null mutant mice is highly affected by their genetic background, leading to early post-natal lethality in a C57BL/6 background but survival to adulthood in an inbred C3H background, which suggests that modifier genes partly compensate for early developmental roles of DSCAM (Fuerst et al., 2010). In *Drosophila*, a third copy of the *Dscam* gene results in sensory perception impairments mirroring those found in flies lacking the Fragile X Mental Retardation gene, in which Dscam levels are elevated, and that in the latter animals can be rescued by reducing *Dscam* expression (Cvetkovska et al., 2013).

On the other hand, *DSCAML1* has been mapped to the 11q23 region, implicated in the pathophysiology of neurodevelopmental disorders including Jacobsen, Gilles de la Tourette, and distal trisomy 11q syndromes which points to *DSCAML1* as a potential causative gene, although a clear causation has not been proven (Agarwala et al., 2001; Pauls, 2003; Mattina et al., 2009; Chen et al., 2014; Choi et al., 2015).

As cell adhesion molecules, DSCAM and DSCAML1 engage in homophilic interactions at the cell membrane, which ensures cell interaction specificity. In arthropods, alternative splicing yields tens of thousands of DSCAM1 isoforms from one gene locus, a process known to be instrumental in achieving self-recognition critical to neural wiring as well as innate immunity (Schmucker et al., 2000; Schmucker and Chen, 2009). This staggering complexity is an insect innovation, as vertebrates can only produce single DSCAM and DSCAML1 isoforms. The higher neural network complexity shown by vertebrate species is thus hypothesized to result from the expansion of other cell adhesion molecule classes with similar characteristics, such as clustered Protocadherins (Jin and Li, 2019).

Previous research in vertebrates and invertebrates has revealed roles for DSCAMs in several neurodevelopmental processes, including synaptogenesis, neural proliferation, dendritic self-avoidance, cell sorting, and axon growth, guidance, and branching (Chen et al., 2006; Fuerst et al., 2008, 2009; Li et al., 2009; Liu et al., 2009; Maynard and Stein, 2012; He et al., 2014; Dascenco et al., 2015; Thiry et al., 2016; Laflamme et al., 2019; Sachse et al., 2019). In the mouse retina, loss of *Dscam* or *Dscaml1* leads to excessive dendritic fasciculation and somatic clustering of the cell types that normally express these molecules, demonstrating a role in dendritic self-avoidance and tiling (Fuerst et al., 2008, 2009). In addition, conditional loss of *Dscam* in the retina produces a decrease in programmed cell death of the targeted population (Fuerst et al., 2012).

Whether these functions are retained and contribute to mammalian forebrain development has yet to be completely elucidated. Research in mouse has shown that *Dscam* loss of function (LOF) results in a transient, early post-natal decrease in the thickness of upper cortical layers; notably, this phenotype could not be attributed to an increase in cell death, nor to a reduction in progenitor proliferation during embryonic development (Maynard and Stein, 2012). Whether the generation of different cortical layers during embryonic brain development is also affected remains unclear. Knockdown of either *Dscam* or *Dscaml1* in the cortex impairs radial migration of projection neurons and leads to a partial mispositioning of presumptive layer II/III neurons in layers IV/V observable for more than 2 weeks after birth. In addition, this partial loss of *Dscam* or *Dscaml1* function in the cortex reduces the midline-oriented extension of callosal axons, which at later post-natal time-points results in a decrease in axon terminals in contralateral cortical regions, supporting the idea that DSCAM and DSCAML1 are important for axon extension, and perhaps also guidance (Zhang et al., 2015). Given the expression of DSCAM and DSCAML1 during embryonic forebrain development, our aim was to further investigate whether these molecules are implicated in the migration of both cortical neurons and interneurons, the patterning/morphogenesis of embryonic telencephalic structures, and the early establishment of forebrain connectivity. Using constitutive loss-of-function models, we demonstrate that loss of DSCAM or DSCAML1 only has minor effects on these processes. However, as in human increased dosage of DSCAM or DSCAML1 seems to be more detrimental to neurodevelopment, we also implemented gain-of-function approaches to study potential roles in neuronal migration and morphological maturation. Our data indicate that overexpression of either DSCAM or DSCAML1 reduced migration distances traveled by immature cortical interneurons, while DSCAML1 overexpression selectively affected neurite branching. Future investigations should reveal the molecular mechanisms at the basis of these phenotypes.

## Materials and Methods

### Animals

All animal procedures were performed in accordance with Belgian and EU regulations on the use of animals for scientific purposes (Royal Decree of 29 May 2013, Directive 2010/63/EU) and approved by the KU Leuven Ethical Committee for Animal Experimentation (project licenses 267/2015 and 005/2017).

All experiments were performed on embryonic brains obtained from C57BL/6J mice (wild-type) (Jackson Laboratories), a *Dlx5/6-Cre-IRES-EGFP* reporter line (Stenman et al., 2003) bred on a CD1 background, a C57BL/6J strain carrying a null mutation in the *Dscam* gene consisting of a 38 bp deletion within exon 17 (*Dscam*^*del*17^) (Fuerst et al., 2008), and a *Dscaml1* null mutant C57BL/6J strain (*Dscaml1*^*GT*^). In the latter case, LOF was achieved by the insertion of a gene-trap vector in the 3rd *Dscaml1* intron, resulting in the production of a non-functional N-terminal DSCAML1–β-galactosidase fusion protein (Fuerst et al., 2009). *Dscam*^*del*17^*; Dlx5/6-Cre-IRES-EGFP* (*Dscam*^*del*17^*; Dlx5/6-CIE*) and *Dscaml1*^*GT*^*; Dlx5/6-Cre-IRES-EGFP* (*Dscaml1*^*GT*^*; Dlx5/6-CIE*) mutant mice were generated by crossing *Dscam*^*del*17^ and *Dscaml1*^*GT*^ lines with the *Dlx5/6-Cre-IRES-EGFP* reporter line. Mouse colonies were maintained in a 14/10 h light-dark cycle, in a humidity- and temperature-controlled pathogen free animal unit.

Pregnant females for embryo collection were obtained via timed matings. Embryonic age was calculated considering the day of vaginal plug detection as E0.5. Mouse brains were dissected in cold phosphate-buffered saline (PBS) and fixed in 4% w/v paraformaldehyde (PFA)/PBS for 16–24 h at 4°C, unless they were processed for X-gal staining. Following fixation, specimens were washed once in PBS for 30–60 min at 4°C, and stored at this temperature for up to 9 months in storage buffer (0.01% w/v thimerosal/PBS). Mouse tail samples (~5 mm) were also collected for DNA extraction and genotyping.

To verify the absence of DSCAM protein in the DSCAM knockout mouse (Supplementary Figure 1I), protein was extracted from E17.5 brains from knockout and wildtype mice using TRIS-HCL SDS-buffer (65 mM Tris-HCL, 2% SDS) containing cOmplete™ Protease Inhibitor Cocktail (Roche). Tissue lysates were cleared by centrifugation and proteins were heat denatured in a mixture of XT sample buffer 4x and XT reducing agent 20x, separated on 4–12% Bis-Tris precast polyacrylamide gels (Criterion XT Bis-Tris Precast Gel, Bio-Rad) in MOPS buffer, and immuno-blotted to nitrocellulose membranes (Trans-Blot Turbo Midi 0.2 μm Nitrocellulose Transfer Packs, Bio-Rad) using a Trans Blot Turbo system (Bio-Rad). Standard protein detection was performed using rabbit anti-DSCAM antibodies (1:250; HPA019234, Sigma-Aldrich). After 2 h blocking in 5% w/v non-fat dry milk/TBST (WB buffer) at RT, o/n incubation at 4°C in primary antibody diluted in WB buffer, and washing in TBST, transfer membranes were incubated for 45 min in HRP-conjugated anti-rabbit secondary antibodies (Bio-Rad) diluted 1:10,000 in WB buffer. Protein bands were visualized with a ChemiDoc MP imaging system (Bio-Rad) after incubation in ECL substrate (Thermo Fisher Scientific).

### Genotyping

Tissue samples were digested overnight (o/n) at 56°C in a 1:100 Proteinase K solution (10 mg/mL in 40% glycerol/nuclease-free H_2_O; Thermo Fisher Scientific) in lysis buffer (50 mM Tris-HCl pH 8.5, 2.5 mM EDTA pH 8, 50 mM NaCl, 1% SDS). Genotyping PCR reactions were prepared using a small aliquot of the digestion solution, a PCR mix (KAPA2G Fast HotStart ReadyMix with dye, KAPA Biosystems) containing dNTPs, a Taq polymerase and a loading dye, and primers for the genes of interest (see Supplementary Table 1).

### *In situ* Hybridization

*In situ* hybridization (ISH) experiments were performed on 20 μm cryosections or 6 μm paraffin sections from E13.5 and E16.5 wild-type brains. To obtain frozen tissue samples, after PFA fixation brains were incubated in 30% sucrose/PBS at 4°C until sinking, submerged in Optimal Cutting Temperature compound (Tissue-Tek, Sakura Finetek) for 1–2 h at 4°C, fast frozen in liquid nitrogen, and maintained at −20°C until sectioning. Paraffin-embedded specimens were first dehydrated by o/n incubation in 50% ethanol/saline at 4°C, then processed for paraffinization (Excelsior AS Tissue Processor, Thermo Fisher Scientific) and embedding (HistoStar Embedding Workstation, Thermo Fisher Scientific). Sectioning of frozen or paraffin-embedded brains was performed with a Microm HM560 cryostat or a Microm HM360 rotary microtome (Thermo Fisher Scientific), respectively; sections were collected on SuperFrost Plus slides (Thermo Fisher Scientific).

Plasmids for the synthesis of antisense *Gad1* riboprobes were a gift from Prof. Brian Condie (University of Georgia) (Maddox and Condie, 2001). *Dscam, Dscaml1* and *Ebf1* ISH probe sequences were amplified from an embryonic cDNA pool with primer pairs 5′-TCAGGAAGTTCACTTGGAACC-3′/5′-TGGAGAATCCCATTCAAGGC-3′ (*Dscam*), 5′-CTTTGTTGTACGAAAGAAGAGGAAG-3′/5′-CATAGATGTCATACTGTCAGCGTTC-3' (*Dscaml1*), and 5′-CAGGAAAGCATCCAACGGAGTGG-3′/5′-GCCCGTGCTTGGAGTTATTGTGG-3′ (*Ebf1*), respectively. Amplicons (521 bp, 747 bp, and 691 bp) were blunt-cloned in pCRII-TOPO vectors using a TOPO TA Cloning Kit (Thermo Fisher Scientific); following transformation of DH5α chemocompetent cells and blue/white screening, successfully transformed colonies were sequenced to determine the quality and orientation of the inserts. Plasmid DNA from selected colonies' cultures was purified using a PureLink HiPure Plasmid Maxiprep Kit (Invitrogen, Thermo Fisher Scientific).

Digoxigenin (DIG)-labeled antisense riboprobes for *Dscam, Dscaml1, Ebf1*, and *Gad1* ISH were produced from plasmid templates linearized overnight at 37°C. An *in vitro* transcription reaction was prepared with 1 μg of linearized plasmid template using a SP6/T7 DIG RNA Labeling Kit (Roche). The synthesized RNA was purified with Micro Bio-Spin P-30 Gel Columns (Bio-Rad) and quantified using a SimpliNano spectrophotometer (Biochrom).

ISH was performed for all section types on a DISCOVERY automated staining platform (Ventana Medical Systems, Roche). Section were first processed for deparaffinization, fixation, pre-treatment, and post-fixation using RiboMap Kit solutions (Roche). The probes of interest were diluted in RiboHybe (Roche) to a final concentration of 150–300 ng/slide, denatured at 90°C for 10 min, and hybridized at 70°C for 6 h. After a series of stringency washes at 68°C in saline-sodium citrate buffer, specifically bound probes were detected by incubation in a 1:1,000 dilution of AP-conjugated sheep anti-DIG antibody in PBS (30 min at 37°C), and visualized using a BlueMap Detection kit (Roche) (7 h substrate incubation at 37°C). At the end of the ISH protocol, all sections were dehydrated in a graded ethanol dilution series (70%, 2 min; 96%, 2 min; 100%, 3 min; 100%, 3 min) and finally washed twice in xylene for 5 min each. Coverslips were applied using Eukitt Quick-hardening mounting medium (Sigma-Aldrich). Brightfield images of the ISH experiments were acquired using a Leica DM6 B microscope connected to a digital CMOS camera (DMC2900, Leica) with the LAS X software suite (Leica). Images were further processed with the Fiji distribution of the open source program Image J (Schindelin et al., 2012) and Adobe Photoshop CC 2018.

### Immunohistochemistry

Vibratome-processed brain sections were stained using a free-floating IHC protocol, in a 12-well plate, and using a shaking platform for all washes/incubations. Serial 60 μm free-floating brain sections were obtained from PFA-fixed brains embedded in 4% w/v agarose/PBS cut with a Microm HM650V vibratome (Thermo Fisher Scientific), and collected in storage buffer. Tissue pre-treatment was then performed by incubation for 1–2 h at room temperature (RT) in a blocking and permeabilization buffer (10% normal donkey serum, 0.3% Triton X-100 in PBS). If heat-induced antigen retrieval was recommended by the manufacturers of the primary antibodies employed, an additional 20–40 min incubation in sodium citrate buffer (10 mM sodium citrate, 0.05% Tween 20, pH 6.0) pre-heated and maintained at 85°C in a hybridization oven, followed by a 20 min cool-down step at RT, was performed before blocking and permeabilization.

Following pre-treatment, the sections were incubated with primary antibodies diluted in storage buffer for 24–48 h at 4°C. Primary antibodies used were rat anti-CTIP2 (1:500, ab18465, Abcam), mouse anti-Islet1 (1:50; 39.4D5, Developmental Studies Hybridoma Bank), rabbit anti Nkx2.1 (1:1,000; sc-13040, Santa Cruz Biotechnology), mouse anti-neurofilament 165 kD (1:100; 2H3, Developmental Studies Hybridoma Bank), chicken anti-GFP (1:1,000; ab13970, Abcam), rabbit anti-TBR1 (1:400; AB10554, Merck-Millipore), mouse anti-SATB2 (1:200; ab51502, Abcam), rabbit anti-RFP (1:2,000; 600-401-379, Rockland Immunochemicals), and mouse anti-HA tag (1:1,000; 6E2, Cell Signaling Technology). The monoclonal anti-neurofilament 165 kD (2H3) and anti-Islet1/2 homeobox (39.4D5) antibodies, developed by respectively by T.M. Jessell and J. Dodd, and by T.M. Jessell and S. Brenner-Morton, were obtained from the Developmental Studies Hybridoma Bank, created by the NICHD of the NIH and maintained at The University of Iowa.

After four 10 min washes in PBS at RT, the sections were subsequently incubated for 2 h at RT, or overnight at 4° C, with donkey-derived secondary antibodies conjugated with Alexa Fluor® dyes (Jackson Immunoresearch or Invitrogen) diluted 1:500 in storage buffer. Next, the tissue samples were washed in PBS at RT in four 10 min cycles, counterstained with 4′,6-Diamidino-2-Phenylindole (DAPI) (Sigma-Aldrich), and finally mounted on SuperFrost Plus slides in Mowiol (Sigma-Aldrich) mounting medium (30% w/v glycerol, 12% w/v Mowiol, 0.1 M Tris-HCl pH 8.5).

Slides were examined with a Leica DM6 B epifluorescence microscope digital CCD camera (DFC365 FX, Leica) or an Olympus FLUOVIEW FV1000 confocal laser scanning microscope. Images acquired using the LAS X or FV10-ASW Viewer v. 4.2c (Olympus) software packages, respectively, and processed as previously described.

### X-Gal Stainings

Whole mount X-gal stainings were performed on freshly dissected brains from *Dscaml1*^*GT*^ mice pre-incubated in X-gal fixative (1% formaldehyde, 0.2% glyceraldehyde, 0.5% Triton X-100 in PBS) at 4°C on a shaker. Incubation time was adjusted according to brain size to respectively, 20 and 35 min for E13.5 and E16.5 specimens. After three 20–30 min washes in cold PBS, pre-fixed brains were subsequently incubated for 24–48 h in the dark at 37°C in freshly prepared staining buffer (5 mM potassium ferrocyanide, 5 mM potassium ferricyanide, 2 mM MgCl_2_, 0.1% Triton-X, 0.01% sodium deoxycholate in PBS) containing 1 mg/mL X-gal (Applichem). Next, samples were repeatedly washed in PBS at 4°C until washout looked completely clear, post-fixed o/n in 4% PFA at 4°C, and vibratome-sectioned as detailed in section Immunohistochemistry. The obtained sections were counterstained with a Nuclear Fast Red–aluminum sulfate 0.1% solution (Sigma), mounted on glass microscope slides, cover-slipped with Mowiol mounting medium, and dried o/n at RT before imaging. Brightfield microscope images were acquired and processed as described in section *in situ* Hybridization.

### Neuroanatomical Tracings

Mixed retrograde and anterograde tracing of reciprocal connections between distinct thalamic nuclei and either the primary visual (occipital) or primary somatosensory (parietal) cortex in wild-type, *Dscam*^*del*17^ and *Dscaml1*^*GT*^ embryonic mouse brains were performed by inserting 0.1–0.3 mm crystals of, respectively, 1,1′-dioctadecyl-3,3,3′,3′-tetramethylindocarbocyanine perchlorate (DiI; Biotium) and 4-(4-(dihexadecylamino)styryl)-N-methylpyridinium iodide (DiA; Biotium) in the superficial cortical layers of E17.5 brain hemispheres, with the aid of a tungsten dissecting probe (World Precision Instruments). Following the insertion of dye crystals, brains were kept in 1% PFA/PBS at RT in the dark for 3–4 weeks to allow diffusion of the carbocyanine tracers in the axonal tracts and thalamic populations of interest.

At the end of their incubation period, brains were vibratome-sectioned as detailed in section Immunohistochemistry. Sections were counterstained with DAPI, mounted in Mowiol mounting medium onto SuperFrost Plus slides, and imaged using the epifluorescence microscope setup also described in section Immunohistochemistry within 48 h after sectioning, to avoid artifacts due to local dye diffusion at the sections' surfaces.

### Expression Plasmid Production and Testing

Expression vectors used in electroporation experiments were synthetized starting from a pCAGGS-IRES-EGFP plasmid backbone (Megason and McMahon, 2002) (a gift from P. Vanderhaeghen, Université libre de Bruxelles), wherein EGFP was replaced by TdTomato. Full length *Dscam* and *Dscaml1* cDNA sequences tagged in frame at the 3′ end with EYFP- and HA tag-encoding sequences were blunt-end cloned into this pCAGGS vector from pcDNA5-FRT-TO-GW-DSCAM-HA, pcDNA5-FRT-TO-GW-DSCAM-EYFP-HA, pcDNA5-FRT-TO-GW-DSCAML1-HA, and pcDNA5-FRT-TO-GW-DSCAM-EYFP-HA plasmids (Sachse et al., 2019) to produce tagged DSCAM/DSCAML1 and tdTomato co-expression constructs. Control pCAGGS vectors were obtained by sub-cloning only EYFP-HA coding sequences. Correct cloning in all novel expression constructs produced was verified by sequencing.

Expression vectors were first tested by transfection in Neuro 2a mouse cells (Sigma). As culture supports, 35 mm glass bottom dishes (Ibidi) pre-coated with Geltrex (Gibco, Thermo Fisher Scientific) at least 3 h before seeding were used for confocal microscopy imaging, while 6-well plates were used for all other applications. 24 h before transfection cells were seeded to a density of 5 × 10^5^ (6-well plate) or 5 × 10^4^ (cell dish) cells, and maintained in a humidified incubator at 37°C using Dulbecco's modified Eagle's medium (DMEM) supplemented with 10% fetal bovine serum (FBS), 2 mM L-Glutamine, and 50 U/mL Penicillin/50 μg/mL Streptomycin (all from Gibco, Thermo Fisher Scientific). On transfection day, cell adherence and confluency were checked under a microscope. Transfection was performed using a Lipofectamine 3000 transfection kit (Thermo Fisher Scientific). Briefly, Lipofectamine 3000 reagent was diluted in Opti-MEM medium (Gibco, Thermo Fisher Scientific) according to manufacturer instructions for 6-well or 24-well (cell dish) plates. 5 μg of plasmid DNA were diluted in Opti-MEM, and subsequently 2 μL of P3000 reagent per 1 μg DNA were added to generate the DNA master mix. The master mix was then combined 1:1 with Opti-MEM-diluted Lipofectamine, and incubated for 30 min at room temperature. The resulting DNA-lipid complex mix was added to each well/dish in volumes recommended by the manufacturer, and cells were re-transferred to a humidified incubator at 37°C. Transfection efficiency was examined after 24 and 48 h under a fluorescent microscope; confocal imaging (see section Immunohistochemistry) of cells cultured on glass bottom dishes was performed 48 h after transfection, following o/n fixation in 4% PFA/PBS at 4°C and washing in storage buffer.

To further verify the synthesis of tagged DSCAM/DSCAML1 proteins, Neuro 2a cells were harvested 48 h post-lipofection and lysed in RIPA buffer (50 mM Tris-HCl pH 8.0, 150 mM NaCl, 1% TritonX-100, 0.5% sodium deoxycholate, 0.1% SDS) containing cOmplete™ Protease Inhibitor Cocktail (Roche). Cell lysates were cleared by centrifugation, and proteins were heat-denatured in Laemmli sample buffer containing 50 mM dithiothreitol, separated on 4–20% polyacrylamide gels (Criterion TGX Stain-Free Protein Gel, Bio-Rad) in Tris-glycine-SDS buffer, and immuno-blotted to nitrocellulose membranes (Trans-Blot Turbo Midi 0.2 μm Nitrocellulose Transfer Packs, Bio-Rad) using a Trans Blot Turbo system (Bio-Rad). Standard protein detection was performed using mouse anti-HA tag antibodies (1:1,000; 6E2, Cell Signaling Technology). After 2 h blocking in 5% w/v non-fat dry milk/TBST (WB buffer) at RT, o/n incubation at 4°C in primary antibody diluted in WB buffer, and washing in TBST, transfer membranes were incubated for 1 h in HRP-conjugated anti-mouse secondary antibodies (Jackson Laboratories or Agilent Technologies) diluted 1:10,000 in WB buffer. Protein bands were visualized with a ChemiDoc XRS+ imaging system (Bio-Rad) after incubation in ECL substrate (Pierce).

### *In utero* Electroporation

*In utero* electroporation (IUEP) of mouse embryonic brains was performed at E14.5 in an aseptic environment. Pregnant females were sedated via intramuscular injection of ketamine (75 mg/kg, Eurovet) and medetomidine (1.0 mg/kg, Orion Pharma), and peri-operative analgesia was provided by a subcutaneous injection of meloxicam (5.0 mg/kg, Boehringer Ingelheim). Once sedation was achieved, an ophthalmic ointment (Terramycin, Pfizer) was applied on the animal's eyes, the abdominal fur was removed, and the exposed skin was disinfected with a povidone-iodine solution. All surgical materials were sterilized using a hot bead sterilizer (FST 250, Fine Science Tools) immediately before laparotomy.

After placing the mouse on a heat mat, two incisions of ~2 cm along the linea alba abdominis were made consecutively through the abdominal skin and the muscle/peritoneum tissue layers. To keep the uterus and peritoneal cavity hydrated, a sterile saline solution pre-heated at 37°C was applied as necessary. The uterine horns were gently pulled out of the abdominal cavity and placed on a sterile gauze. 2 μg/μL solutions of DSCAM-EYFP-HA, DSCAML1-EYFP-HA, or EYFP-HA expression constructs diluted in Opti-MEM medium, supplemented at a 1:30 ratio with a Fast Green FCF dye solution (1 mg/mL, Sigma-Aldrich) for visualization purposes, were micro-injected in the embryo's lateral ventricles with glass microcapillary needles (Harvard Apparatus) produced with a magnetic puller (PN-31, Narishige), and connected to a filtered aspirator tube assembly (Drummond). Following bilateral injections, CUY650P5 tweezer electrodes connected to a NEPA21 electroporator (Nepa Gene) were washed with saline solution and positioned at the sides of the embryo's head for electroporation (see Supplementary Table 2 for IUEP parameters) (**Figure 6A**). The injection and electroporation steps were repeated for a maximum of 8 embryos per female.

At the end of the procedure, the uterus was re-positioned within the abdominal cavity, and the abdominal incisions were closed using non-absorbable suture (PERMA-HAND silk, Ethicon). A povidone-iodine solution and an antibiotic cream (Fucidin, Leo Pharma) were applied on the sutured wound, and atipamezole (0.5–1.0 mg/kg, Orion) was finally injected intramuscularly to reverse anesthesia. After the surgery, mice were allowed to recover o/n in cages placed on a heating pad at 37°C, and provided with fresh bedding material, food and water. All operated pregnant females were kept in the animal facility until E18.5, when they were sacrificed for embryonic brain collection.

To better detect the transfected neurons in the obtained brains, free-floating IHC using primary antibodies against tdTomato and EYFP, and secondary antibodies matching the excitation/emission spectrum of the respective fluorescent protein, were performed on coronal vibratome sections from the electroporated brains before confocal imaging. Immunohistochemistry was performed as described in section Immunohistochemistry. Confocal imaging equipment and procedures are detailed in section Immunohistochemistry and Phenotype Quantification and Statistical Analysis.

### Medial Ganglionic Eminence Electroporation and Explant Culture

Medial ganglionic eminence (MGE) electroporation (MEP) and MGE explant cultures were performed under sterile conditions on E13.5 brain tissue from *Dlx5/6-Cre-IRES-EGFP* mouse embryos. Pregnant females were euthanized by cervical dislocation to collect E13.5 embryos, the heads of which were dissected in cold Leibovitz's L-15 medium (Gibco, Thermo Fisher Scientific) supplemented with 35 mM D-glucose (Merck Millipore) and 2.5 mM HEPES (Gibco, Thermo Fisher Scientific) (L15++). For each isolated EGFP+ embryonic head, the MGEs were exposed by incisions at dorsal cortical level, and 2 μg/μL solutions of overexpression or control plasmids were injected with in 8–10 discrete MGE sites per hemisphere; next, injected brains were electroporated with CUY650P5 tweezer electrodes connected to a BTX electroporator (Harvard Apparatus) (see section *in utero* Electroporation for solution composition and injection material details, and Supplementary Table 3 for MEP parameters) (**Figure 7A**).

Electroporated heads were left in L15++ medium for a minimum of 3 h on ice to allow recovery of neural cells. Subsequently, each MGE was dissected under a stereomicroscope to obtain ~8 similarly sized explants (~400–500 μm of diameter), which were transferred to cold Neurobasal Medium containing 2.5 mM HEPES, 2 mM L-glutamine, 100 U/mL Penicillin-Streptomycin, 1x B-27 supplement (all from Gibco, Thermo Fisher Scientific) (Complete Neurobasal Medium, CNB). Each explant was embedded on ice in ~20 μL of Matrigel (Corning Life Sciences) diluted 1:1 in CNB, using as a support 35 mm glass bottom cell culture dishes (Ibidi). Embedded explants were briefly incubated at 37°C to enable Matrigel polymerization, covered with 500 μL of CNB, and cultured for 48 h at 37°C, 5% CO_2_ in a humidified incubator (**Figure 7A**). Finally, explant cultures were fixed o/n in 4% PFA/PBS at 4°C, and preserved in storage buffer until imaging.

### Phenotype Quantification and Statistical Analysis

For cortical lamination and thickness analyses in E17.5 *Dscam*^*del*17/*del*17^, *Dscaml1*^*GT*/*GT*^, or wild-type brains, cell counts per marker and radial measurements were obtained from single plane confocal images of three sections (representative of rostral, intermediate and caudal positions on the rostro-caudal axis) per specimen with the ImageJ Cell Counter plugin and Measure function. Data was statistically analyzed via a mixed ANOVA test, with rostro-caudal position as a within-subject factor and genotype as a between-subject factor, using Greenhouse-Geisser corrections if the assumption of sphericity was violated.

Interneuron distribution along the cortical radial axis of E18.5 *Dscaml1*^*GT*/*GT*^; *Dlx5/6-CIE* and *Dscaml1*^+/+^; *Dlx5/6-CIE* brains was assessed following IHC against eGFP, on coronal sections at a rostral and caudal level. In total three *Dscaml1*^*GT*/*GT*^; *Dlx5/6-CIE* and two *Dscaml1*^+/+^; *Dlx5/6-CIE* brains were analyzed, each including two individual technical replicates. A rectangle of 200μm x 550μm (rostral) and 200 × 500 μm (caudal) in the same medio-lateral region in the cortex was delineated, and further divided into 10 bins (bin1 = pial to bin10 = ventricular) of equal size using Fiji. Integrated density was quantified per bin, and normalized for area.

Radial migration following IUEP was assessed by measuring the distribution of TdTomato fluorescence along the cortical radius in confocal images (4 μm step maximum Z-stacks projections) of coronal brain sections. TdTomato fluorescence intensity values were acquired within 200 um dorso-lateral cortex sectors, divided in 10 equal bins, with the ImageJ Plot Profile function. A mixed model ANOVA test with bin as a within-subject factor and expression construct as a between-subject factor was carried out to compare means per bin across treatment groups. Greenhouse-Geisser corrections were applied when the assumption of sphericity was violated by data.

MGE explant migration was quantified by measuring linear distances from explant edge of TdTomato+ neurons on mixed brightfield-fluorescence confocal images (2 μm step maximum Z-stacks projections) (**Figures 7B–E**) with the ImageJ Measure function. Morphological analysis of MGE explant-derived neurons was performed using the SNT ImageJ plugin (Longair et al., 2011; Arshadi et al., 2020). A minimum of 9 explants obtained from at least two experimental replicates were analyzed per treatment group. Means per experimental group were compared with a one-way ANOVA followed by a Tukey *post-hoc* test. If data did not meet basic requirements for use with parametric models, a Kruskal–Wallis one-way ANOVA on ranks followed by Dunn's pairwise tests with Bonferroni adjustments were employed instead. Frequencies per neuron category were compared across experimental conditions with a Pearson's Chi-square test followed by a *post-hoc* residuals analysis, applying a Bonferroni correction for multiple comparisons.

## Results

### *Dscam* and *Dscaml1* Are Dynamically Expressed During Embryonic Forebrain Development

*Dscam* and *Dscaml1* expression patterns in the mouse telencephalon during embryonic development have been so far poorly characterized. Thus, to better understand in which cellular and neurostructural context they might provide crucial functions, the spatiotemporal dynamics of *Dscam* and *Dscaml1* expression were first analyzed by *in situ* hybridization and X-gal staining.

**Figure 1:**
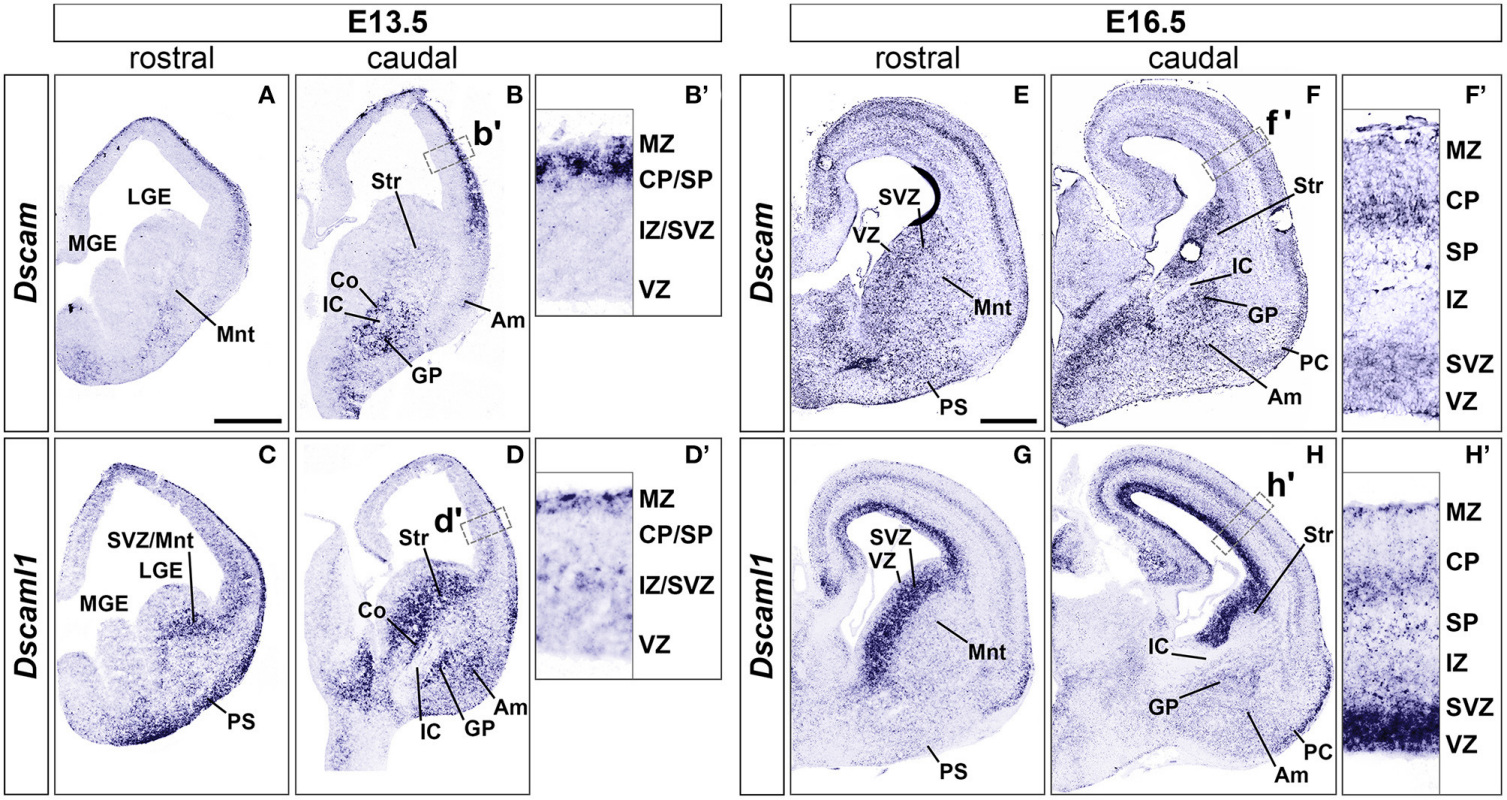
Expression of *Dscam* and *Dscaml1* mRNA in the developing mouse forebrain. Levels of mRNA expression were detected by ISH with antisense RNA probes. **(A–D)** *Dscam* and *Dscaml1* expression in coronal sections of wild-type E13.5 mouse brains. **(A,B)** *Dscam* is restrictedly expressed in post-mitotic regions of both E13.5 dorsal and ventral telencephalon across the rostro-caudal axis. In the subpallium, high transcription levels are specifically observed in structures surrounding the IC, corresponding to the corridor and the GP; sparse expression is also detected throughout the mantle region, in presumptive striatal and amygdalar territories **(B)**. Within the developing cortex, *Dscam* mRNA is highly present in the MZ and upper CP **(B')**. **(C,D)** *Dscaml1* transcription at E13.5 occurs mostly in post-mitotic areas of the dorsal and ventral telencephalon, as well as subventricular progenitor zones. Robust expression is observed in territories where *Dscam* mRNA is also present, e.g., in subpallial cell populations of the corridor, GP, presumptive striatum and amygdala, and is additionally detected in the SVZ (D). In the dorsal pallium, *Dscaml1* mRNA is observed in some cells of the MZ and IZ/SVZ **(D')**. **(E-H)** *Dscam* and *Dscaml1* expression in coronal sections of wild-type E16.5 mouse brains. **(E,F)** At E16.5, *Dscam* remains robustly, but sparsely expressed in subpallial areas surrounding the IC; transcription furthermore extends to the pial surface ventrally, and the VZ/SVZ dorsally across the rostro-caudal axis. In this latter region, expression reaches high levels comparable to those found in IC-adjacent areas **(F)**. In the dorsal forebrain, *Dscam* mRNA is detected in sparse cells occupying most cortical layers; highest transcription levels can be observed in the CP and the SVZ **(F')**. *Dscam* expression can also be observed at PC level. **(G,H)** In the E16.5 telencephalon, *Dscaml1* shows maximum subpallial expression in the SVZ; sparse cells presenting moderate transcription can be additionally detected ventrally to this region, particularly in GP, presumptive amygdala, and presumptive striatum populations **(H)**. At cortical level, *Dscaml1* mRNA is detected at high levels in most cells of the VZ and SVZ; sparse cells showing robust transcription can be observed throughout the radial extension, with a higher accumulation at the SP, CP, and MZ **(H')**. Am, amygdala; Co, corridor; CP, cortical plate; GP, globus pallidus; IC, internal capsule; IZ, intermediate zone; LGE, lateral ganglionic eminence; MGE, medial ganglionic eminence; Mnt, mantle; MZ, marginal zone; PC, piriform cortex; PS, pial surface; SP, subplate; Str, striatum; SVZ, subventricular zone; VZ, ventricular zone. Scale: **(A–D)**, 500 μm; **(E–H)**, 500 μm.

At embryonic day (E) 13.5 (*n* = 5), *Dscam* was found to be strongly expressed in postmitotic layers of the developing cortex [the marginal zone (MZ) and the preplate (PP)/cortical plate (CP)], and in mantle regions of the ventral telencephalon (vTel) surrounding the internal capsule (IC), comprising the subpallial corridor dorsally and the globus pallidus ventrally. Sparse transcription was also observed in the presumptive striatum and amygdala, and in pial surface layers (Figures 1A,B). At E16.5 (*n* = 5), *Dscam* expression appeared to have extended to all cortical layers, except for the ventricular zone (VZ), and was found to be particularly robust in deeper cortical plate regions. Similarly, in the E16.5 vTel *Dscam* mRNA could also be clearly detected in progenitor zones, particularly the subventricular zone (SVZ), and additionally in the piriform cortex (Figures 1E,F).

Concerning *Dscaml1* expression, ISH at E13.5 (*n* = 5) revealed similar transcriptional patterns to those of *Dscam*; however, high mRNA levels were additionally observed in subventricular layers of both cortex and vTel, and expression at the cortical MZ and the vTel pial surface appeared stronger and denser at more rostral levels (Figures 1C,D). At E16.5 (*n* = 5), high transcriptional activity was still observed in both cortical and subpallial SVZs, and vTel ventral surface regions corresponding to the piriform cortex and cell populations delineating the lateral olfactory tract (LOT); sparse expression could be further detected at the VZs, the subplate, the deep CP, the MZ, the vTel VZ, the striatum, and the amygdala (Figures 1G,H). These findings align with expression patterns collected in the Allen Developing Mouse Brain Atlas (Thompson et al., 2014).

In the absence of sufficiently specific and sensitive antibodies against DSCAML1 epitopes for IHC purposes, DSCAML1 protein expression patterns in the developing mouse forebrain were investigated by whole mount X-gal staining on *Dscaml1*^+/*GT*^ and *Dscaml1*^*GT*/*GT*^ brains at E13.5 and E16.5 (*n* = 3 per genotype for both time-points). In these mice, the insertion of a gene-trap vector in the 3rd *Dscaml1* intron resulted in the production of a non-functional N-terminal DSCAML1–β-galactosidase fusion protein (Fuerst et al., 2009). While overlapping domains of protein synthesis and mRNA expression were found, there were also significant discrepancies in their pattern. At E13.5, X-gal stainings highlighted translation only in a few areas where transcription was detected by ISH, namely the MZ, the pial surface of the vTel, the pallidum, and a vTel mantle region delineating the pallial-subpallial boundary (Supplementary Figures 1A–D). Similarly, E16.5 samples indicated protein production resembling mRNA expression in the dorsal pallium, but distinct in the caudate-putamen, the amygdala, demonstrating remarkably high levels in areas surrounding the LOT, in particular the nucleus of the LOT (nLOT), the anterior and central amygdaloid areas, and the cortical, medial, and basolateral amygdaloid nuclei (Supplementary Figures 1E–H). Whether this is due to differences in protein half-life and/or post-transcriptional regulation remains to be determined.

### Striatal Development Occurs in an Overall Normal Manner in *Dscaml1* Null Mutants

**Figure 2:**
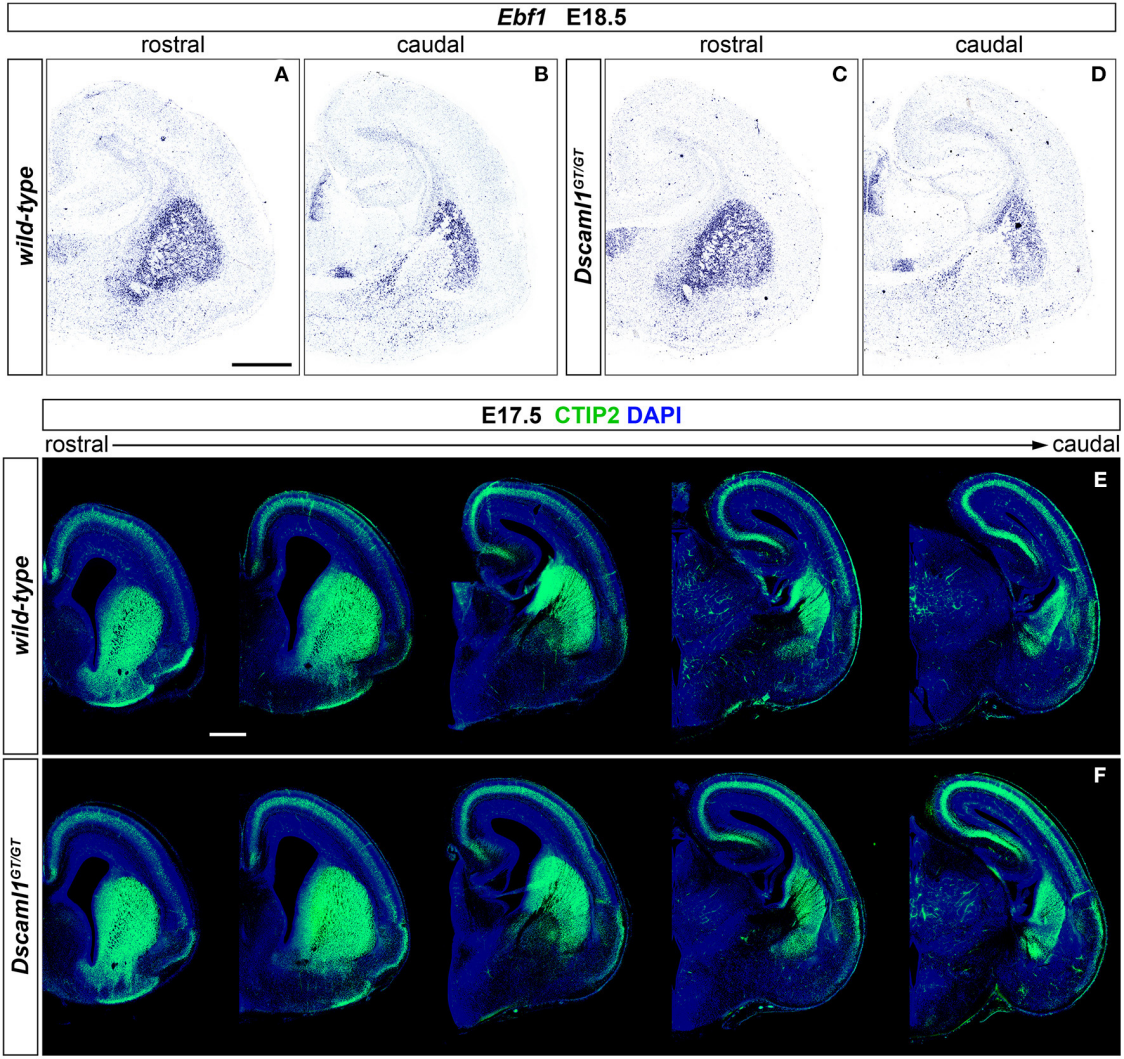
Normal striatal development in embryonic *Dscaml1* null mutant mouse brains. **(A–D)** *Ebf1* mRNA expression, detected by ISH with antisense RNA probes, in coronal sections of wild-type and *Dscaml1*^*GT*/*GT*^ E18.5 mouse brains. Spatial expression patterns of *Ebf1*, a marker of striatal postmitotic neurons mostly populating the matrix compartment, detected in Dscaml1GT/GT specimens **(C,D)** are comparable to those observed in wild-type brain tissue **(A,B)** across the rostro-caudal axis. In both cases, *Ebf1*-expressing neurons span the ventral SVZ and mantle regions of the vTel in a compact manner rostrally **(A,C)**, and delineate the corridor region dorsal to the IC caudally **(B,D)**. Moreover, ISH results highlight the preservation of the striatosome/matrix cytoarchitecture in the striatum of *Dscaml1*^*GT*/*GT*^ animals (A), as compared to wild-type mice **(C)**. **(E,F)** IHC for the striatal medium spiny neuron marker CTIP2 (green) on coronal E18.5 brain sections reveals similar patterns of expression between wild-type (E) and *Dscaml1*^*GT*/*GT*^ embryos **(F)**. As expected in normal development, *Dscaml1*^*GT*/*GT*^ CTIP2+ neurons are present at high density in SVZ and mantle subpallial regions; at intermediate rostro-caudal levels, CTIP2+ cells populate the corridor region, while being mostly absent from the GP. Scale: 500 μm.

Expression of *Dscaml1* seemed prominent in the ventral telencephalon (Figures 1C,D and Supplementary Figures 1A–H). To test whether the development of striatal cell populations and the striatal cytoarchitecture were affected by loss of *Dscaml1* function, the expression patterns of distinct striatal neural markers were examined in *Dscaml1*^*GT*/*GT*^ vs. wild-type embryonic brain at E17.5 and E18.5, which corresponds to a peak in SVZ-specific matrix neurogenesis (van der Kooy and Fishell, 1987; Hamasaki et al., 2001). The first marker analyzed was Ebf1, a transcription factor predominantly labeling postmitotic neurons of the matrix component, and providing essential functions in normal striatal development (Garel et al., 1999; Lobo et al., 2008; Faedo et al., 2017; Tinterri et al., 2018). ISH with *Ebf1* mRNA-specific antisense probes revealed that at E18.5 (*n* = 4 per genotype) expression of this striatal marker occurred comparably in wild-type (Figures 2A,B) and Dscaml1GT/GT (Figures 2C,D) brains; transcription appeared preserved throughout postmitotic neurons of the caudate-putamen region in *Dscaml1*^*GT*/*GT*^ specimens, and, like in wild-type sections, concentrated in the striatal matrix compartment. Moreover, at dorsal vTel level, in the SVZ/upper mantle area, neurons expressing *Ebf1* at E18.5 delineated a compact cell layer in both wild-type and *Dscaml1*^*GT*/*GT*^ rostral brain sections, suggesting a correct distribution of striatal neurons at the site where loss of X-gal staining in *Dscaml1*^*GT*/*GT*^ was previously detected.

Since Ebf1 represents a striatal subpopulation-specific cell fate marker, protein synthesis patterns of CTIP2, a transcription factor crucially involved in medium spiny neuron (MSN) differentiation and striatal cytoarchitecture establishment (Arlotta et al., 2008; Tinterri et al., 2018), were further investigated to acquire a broader view of caudate-putamen development in *Dscaml1*^*GT*/*GT*^ embryonic brains. Comparison of immunostainings on E17.5 brain sections from wild-type and *Dscaml1*^*GT*/*GT*^ specimens (*n* = 4 per genotype) (Figures 2E,F) indicated a normal spatiotemporal transcription of CTIP2 of the *Dscaml1*^*GT*/*GT*^ vTel, and a proper distribution of MSNs in the dorsal striatum with *Dscaml1* LOF.

Based on analysis of striatal markers Ebf1 and CTIP2, no gross abnormalities could be detected in the embryonic development of caudate and putamen nuclei in DSCAML1-deficient mouse brains.

### Subpallial Cytoarchitecture and Internal Capsule Tracts Remain Properly Established With *Dscam* or *Dscaml1* Loss of Function

ISH and IHC experiments highlighted the expression of both *Dscam* and *Dscaml1* in both pallial and subpallial domains delineating pathways where IC axonal tracts, including cortical-subcortical connections such as the thalamocortical and corticothalamic axons (TCA and CTA), elongate during embryonic development. These projections extend in the vTel starting as early as E11.5 until ~E15.5, supported in their navigation by intermediate subpallial targets expressing critical guidance cues, such as the corridor region and the striatum (Auladell et al., 2000; Molnár et al., 2012; Garel and López-Bendito, 2014). The presence of DSCAM and DSCAML1 in these structures therefore hinted at possible roles in the embryonic establishment of forebrain connectivity, likely by contributing to vTel morphogenetic processes.

**Figure 3:**
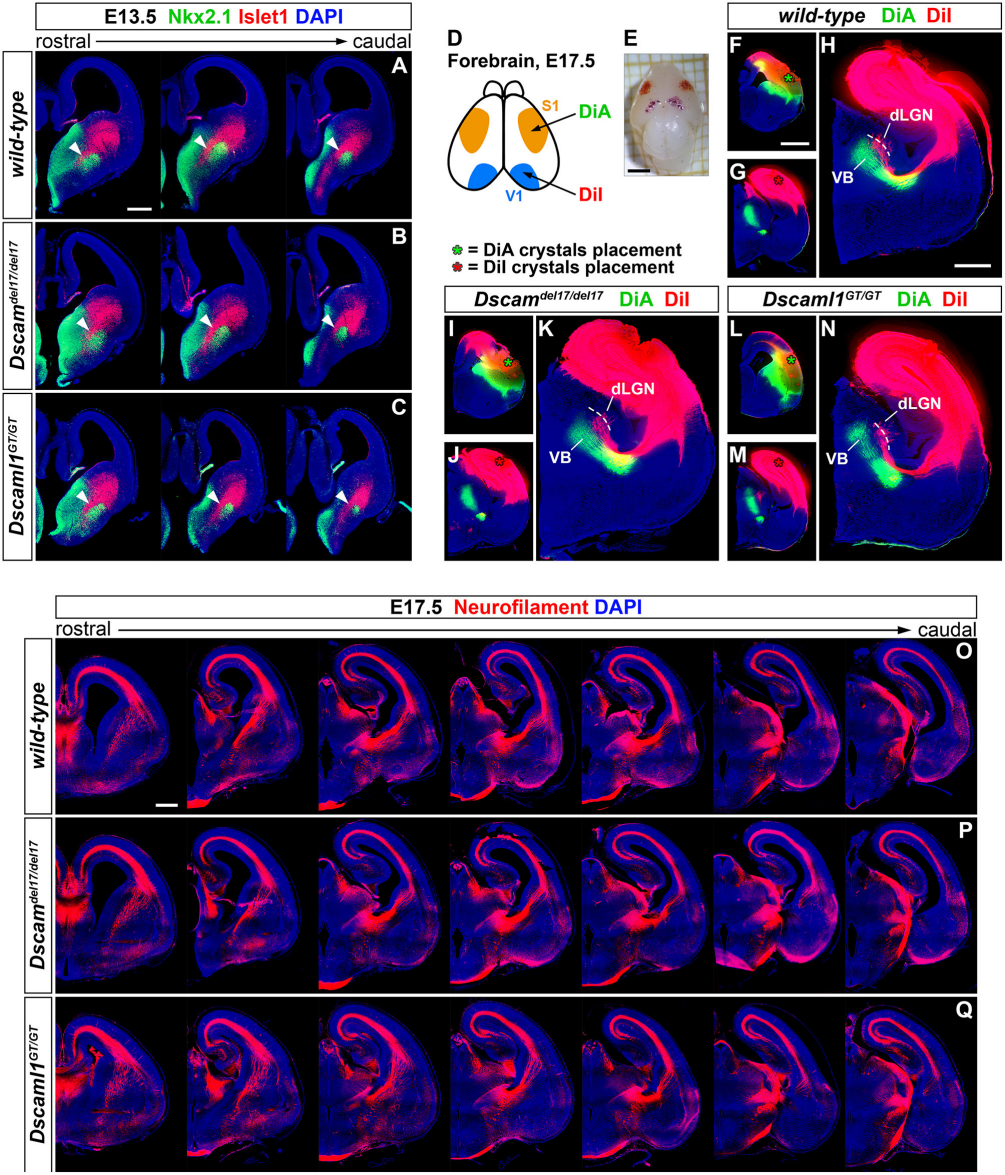
Preserved subpallial cytoarchitecture and IC connectivity in embryonic *Dscam* and *Dscaml1* null mutant mouse brains. **(A–C)** Double IHC for Nkx2.1 (green) and Islet1 (red) on E13.5 coronal brain sections indicates the normal organization, at this stage, of Islet1+ and Nkx2.1+ cell domains in the developing vTel of *Dscam*^*del*17/*del*17^ **(B)** and *Dscaml1*^*GT*/*GT*^ **(C)** mouse brains, as compared to wild-type specimens **(A)**. LGE-derived, Islet1-expressing neurons can be observed sparsely in the SVZ, and at high density throughout the striatum; Islet1+ cells additionally form a narrow band (i.e., the subpallial corridor; white arrowheads) dorsally adjacent to the GP, which is characterized by cells derived from MGE progenitors and thus expressing Nkx2.1. **(D–N)** Neuroanatomical tracing of TCAs and CTAs with two distinct carbocyanine dyes from the visual (occipital) and the somatosensory (parietal) cortex areas in E17.5 wild-type, *Dscam*^*del*17/*del*17^, and *Dscaml1*^*GT*/*GT*^ mouse brains. **(D,E)** Schematic representation of cortical dye placement sites in E17.5 brain hemispheres **(D)**. DiA (green) and DiA (red) crystals are placed respectively within parietal (S1) and occipital (V1) regions of the cortex, as shown in the sample brain illustrated **(E)**. **(F–N)** Insertion of DiA crystals in parietal cortical areas of wild-type **(F)**, *Dscam*^*del*17/*del*17^ **(I)**, and *Dscaml1*^*GT*/*GT*^ **(L)** brains results in retrograde labeling of thalamic neurons of the ventro-medial VB nucleus comparably across all genotypes examined **(H,K,N)**. Likewise, retrograde tracing from parietal cortical sites in wild-type **(G)**, *Dscam*^*del*17/*del*17^ **(J)**, and *Dscaml1*^*GT*/*GT*^ **(M)** brains using DiI crystals leads to similar labeling of thalamic neurons in dLGN and dorso-lateral VB in all samples analyzed **(H,K,N)**. **(O–Q)** Immunostaining for neurofilament (NF; red) in coronal E17.5 brain sections confirms the absence of gross abnormalities in IC tracts' development and morphology in either *Dscam*^*del*17/*del*17^ **(P)** or *Dscaml1*^*GT*/*GT*^ mutant embryos **(Q)**, as compared to wild-type specimens **(O)**. NF+ TCAs and CTAs traverse the diencephalic-telencephalic boundary in a tight bundle, within the IC, spread in a fan-like shape at striatal level, and compactly elongate within the cortex in the IZ after crossing the pallial-subpallial boundary. dLGN, dorsal lateral geniculate nucleus; VB: ventrobasal complex. Scale: **(A–C)**, 200 μm; **(E)**, 2 mm; **(F,G,I,J,L,M)**, 1 mm; **(H,K,N)**, 600 μm.

To first test this hypothesis, the correct formation of subpallial territories allowing TCAs and CTAs to proceed toward the cortex within the IC was investigated by double IHC for the transcription factors Islet1 and Nkx2.1. At intermediate stages of TCA and CTA development (E12–E15), these proteins mark two distinct vTel populations, the LGE-derived corridor and striatal cells and the MGE-derived globus pallidus neurons, respectively permissive and repellent to TCA growth (López-Bendito et al., 2006). IHC performed on wild-type, *Dscam*^*del*17/*del*17^, and *Dscaml1*^*GT*/*GT*^ brain sections at E13.5 (*n* = 3 per genotype) (Figures 3A–C), when corridor neurons are expected to have fully migrated from the LGE to the MGE mantle area dorsal to the globus pallidus, demonstrated similar Islet1 and Nkx2.1 immunostaining patterns between wild-type and mutant specimens. At IC level, Islet1 was detected in a narrow band of cells lining a pathway for TCAs between the Nkx2.1+ globus pallidus and the SVZ of the MGE, corresponding to the proper location of corridor neurons at this developmental stage. Additionally, immunostaining could be clearly observed in LGE-derived striatal regions. Thus, Islet1/Nkx2.1 IHCs provided evidence for an appropriate cytoarchitectural development and cellular differentiation of subpallial territories required for TCA and CTA axon guidance.

While proper vTel morphogenesis was observed in *Dscam* and *Dscaml1* knockout mice, it could not be excluded that the function of both corridor and striatal territories might be altered in these animals, and thus still give rise to topographical IC axonal sorting issues. Moreover, the presence of both DSCAM molecules in the developing cortex suggested potential TCA navigation and targeting functions at pallial level. To explore the possibility of reciprocal connectivity alterations between neocortical areas and dorsal thalamic nuclei arising due to defects in TCA/CTA guidance with *Dscam* or *Dscaml1* LOF, targeted axonal tracing experiments were performed in wild-type and mutant mouse brains at late embryonic development stages, at which major axonal tracts have been established. Mixed retrograde and anterograde double-tracing experiments were carried out by placing crystals of the carbocyanine dyes DiI and DiA in, respectively, occipital and parietal cortical areas of *Dscam*^*del*17/*del*17^, *Dscaml1*^*GT*/*GT*^, and wild-type E17.5 mouse brains (*n* = 3 per genotype) (Figures 3D–N). Neuroanatomical tracings indicated that connectivity between different TCA subsets and their cognate cortical domains is preserved in both *Dscam*^*del*17/*del*17^ and *Dscaml1*^*GT*/*GT*^ mice. Like in wild-type mouse brains, DiA crystals placed in the parietal cortex of knockout specimens, at the level of somatosensory areas, led to the back-labeling of a medial ventrobasal complex (VB) cell population. Furthermore, DiI crystals placed in the occipital cortex, diffusing within visual and auditory processing regions, retrogradely traced somas in a dorsal VB neuronal subset and within the dorsal lateral geniculate nucleus in both wild-type and mutant brains.

To better investigate the formation and spatial organization of IC axonal tracts in mouse brains lacking *Dscam* or *Dscaml1*, IHC for the 165 kDa neurofilament (NF) subunit, a pan-axonal marker, was additionally performed on *Dscam*^*del*17/*del*17^, *Dscaml1*^*GT*/*GT*^, and wild-type E17.5 brain sections (*n* = 3 per genotype) (Figures 3O–Q). Consistently with previous findings, TCAs and CTAs were observed to correctly navigate the vTel in both *Dscam* and *Dscaml1* knockout forebrains, traversing the medial subpallium in a tight axonal bundle (i.e., the IC), while extending in a fan-like shape in the lateral subpallium. At cortical level, thalamocortical and corticofugal projections elongated in a compact tract within the IZ, as normally expected. Moreover, the examination of striatonigral and nigrostriatal connections, which also express NF (Uemura et al., 2007) and elongate within the IC, revealed a preserved spatial navigation of other IC projections with *Dscam* and *Dscaml1* LOF.

### Interneuron Migration Is Grossly Preserved in Developing Brains Lacking *Dscam* or *Dscaml1*

**Figure 4:**
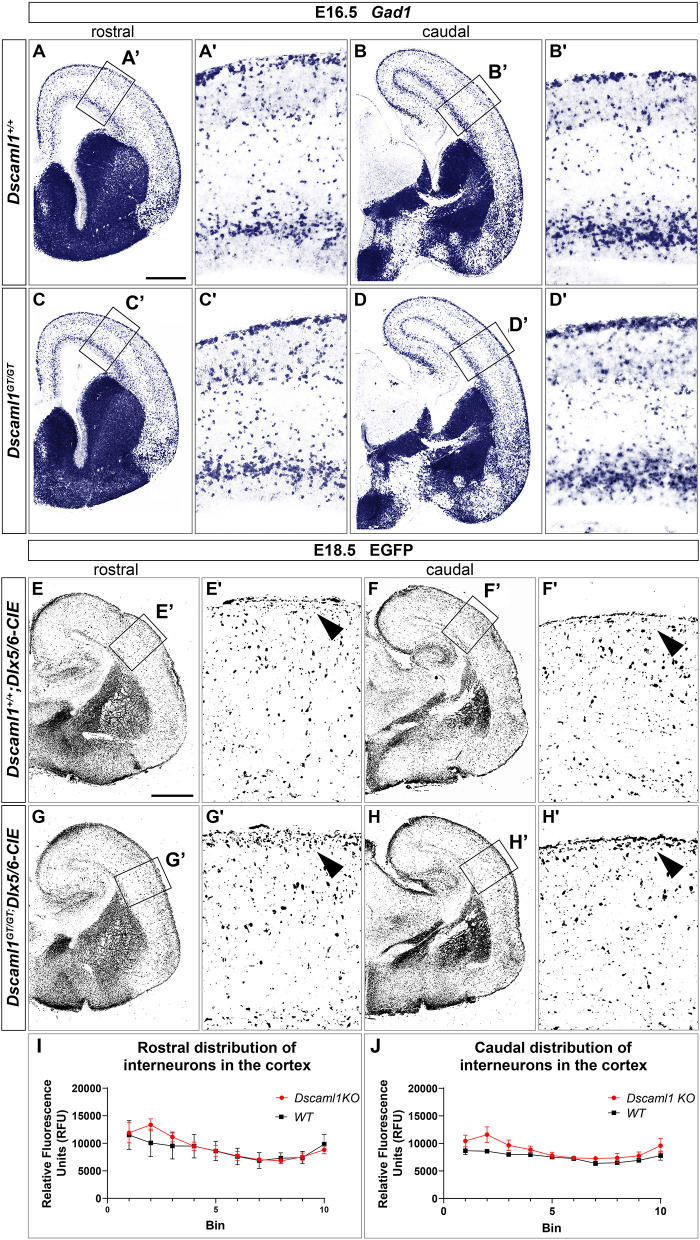
Interneuron migration in *Dscaml1* null mutant mouse brains. **(A-D)** *Gad1* mRNA expression, detected by ISH with antisense RNA probes, in coronal sections of wild-type **(A,B)** and *Dscaml1*^*GT*/*GT*^ E16.5 **(C,D)** mouse brains. In both cases, *Gad1*-expressing neurons can be observed from SVZ to pial surface regions of the vTel; at the pallial-subpallial boundary, labeled GABAergic cortical interneurons tangentially invade the developing cortex in two main streams within the SVZ/IZ and MZ. At this developmental stage, cortical INs spread radially within the cortex toward their presumptive target layers, and can be found across all cortical laminae, particularly in the CP. *Gad1*-expressing cell densities and distribution patterns within the cortex are comparable between genotypes **(A'–D')**. **(E–H)** IHC using anti-EGFP antibodies on coronal sections from *Dscaml1*^+/+^; *Dlx5/6-CIE* and *Dscaml1*^*GT*/*GT*^; *Dlx5/6-CIE* E18.5 brains. EGFP-labeled cortical interneurons, derived from subpallial territories expressing *Dlx5/6*, similarly distribute in the cortex of wild-type **(E,F)** and *Dscaml1*^*GT*/*GT*^ mutant specimens **(G,H)** in both tangential and radial directions. An accumulation of *Dlx5/6*-labeled neurons is observable at MZ level in *Dscaml1*^*GT*/*GT*^ brain sections compared to wild-type sections [**(E'–H')**, black arrowheads]. **(I,J)** Quantification of interneuron distribution on coronal sections from *Dscaml1*^+/+^*; Dlx5/6-CIE* and *Dscaml1*^*GT*/*GT*^*; Dlx5/6-CIE* E18.5 brains. In both rostral **(I)** and caudal **(J)** sections, EGFP-labeled interneuron accumulation is confined to Bin 2 (with Bin 1 and 10 corresponding, respectively, to the most superficial and deeper radial bins the cortex is divided into) in Dscaml1GT/GT brains compared to wild-type brains. Scale: **(A–D)**, 500 μm; **(E–H)**, 500 μm.

ISH and X-gal experiments highlighted the presence of DSCAM and DSCAML1 in forebrain regions corresponding to neural territories where immature cortical interneurons (INs) originate (e.g., the subpallial SVZ and mantle) or migrate into (e.g., the cortical MZ and IZ) during embryonic development. To investigate whether *Dscam* or *Dscaml1* LOF affects the navigation of GABAergic neurons toward their cortical targets, INs were first studied in *Dscam*^*del*17/*del*17^ and *Dscaml1*^*GT*/*GT*^ vs. wild-type embryonic brains via ISH with DIG-labeled antisense *Gad1* probes. ISH experiments were performed at E13.5 in *Dscam*^*del*17/*del*17^ brains, and at E16.5 in *Dscaml1*^*GT*/*GT*^ brains, based on X-gal staining results suggesting the absence of DSCAML1 transcription in most subpallial and cortical areas around E13.5. ISH results indicated that the absence of DSCAM does not affect cortical IN generation or population of the cortex by cortical IN: at E13.5, *Gad1*-expressing cells were abundantly found as normally expected in post-mitotic regions of the subpallium, and from this area MZ and SVZ/IZ streams of migrating INs could be clearly detected in both *Dscam*^*del*17/*del*17^ and wild-type coronal brain sections (Supplementary Figures 2A,B). These streams could be observed to extend tangentially within the developing cortex to a comparable degree and density in *Dscam*^*del*17/*del*17^ and wild-type specimens (*n* = 3 per genotype). At E16.5, loss of *Dscaml1* also did not seem to impair IN entry in the dorsal pallium (Figures 4A–D), as *Gad1*-expressing cells were detected throughout post-mitotic vTel territories, and delineated MZ and SVZ/IZ streams of INs extending uniformly from the subpallium to the cortical hem. Within the cortex, GABAergic neurons were found at high densities within the aforementioned streams, and in addition more sparsely across the cortical radius, in particular within the CP layer.

As *Dscaml1* expression occurs robustly in the vTel SVZ (a progenitor zone for cortical INs) throughout later stages of embryonic development, and is also maintained at the level of both cortical IN streams, GABAergic cell migration was further investigated at a peri-natal developmental time-point, E18.5, in *Dscaml1*^*GT*/*GT*^*; Dlx5/6-Cre-IRES-EGFP* (*Dscaml1*^*GT*/*GT*^; *Dlx5/6-CIE)* and *Dscaml1*^+/+^; *Dlx5/6-Cre-IRES-EGFP (Dscaml1*^+/+^*; Dlx5/6-CIE)* mutant mice, in which cortical INs are endogenously labeled by EGFP. IHC for EGFP on coronal brain sections (Figures 4E–H) confirmed previous ISH findings at E16.5: in *Dscaml1* null mutant specimens, GABAergic INs reached the cortex, and were distributed similarly to the control within the cortical field. However, compared to wild-type, INs were found to accumulate more densely within the MZ in *Dscaml1*^*GT*/*GT*^ mutant brains at E18.5 (Figures 4E–H, arrowheads, quantification in Figures 4I,J). Thus, findings overall indicated that *Dscaml1* LOF might subtly affect the distribution of cortical INs close to the marginal zone at late embryonic developmental stages.

### Embryonic Cortical Development and Lamination Are Unaffected by *Dscam* or *Dscaml1* Loss of Function

**Figure 5:**
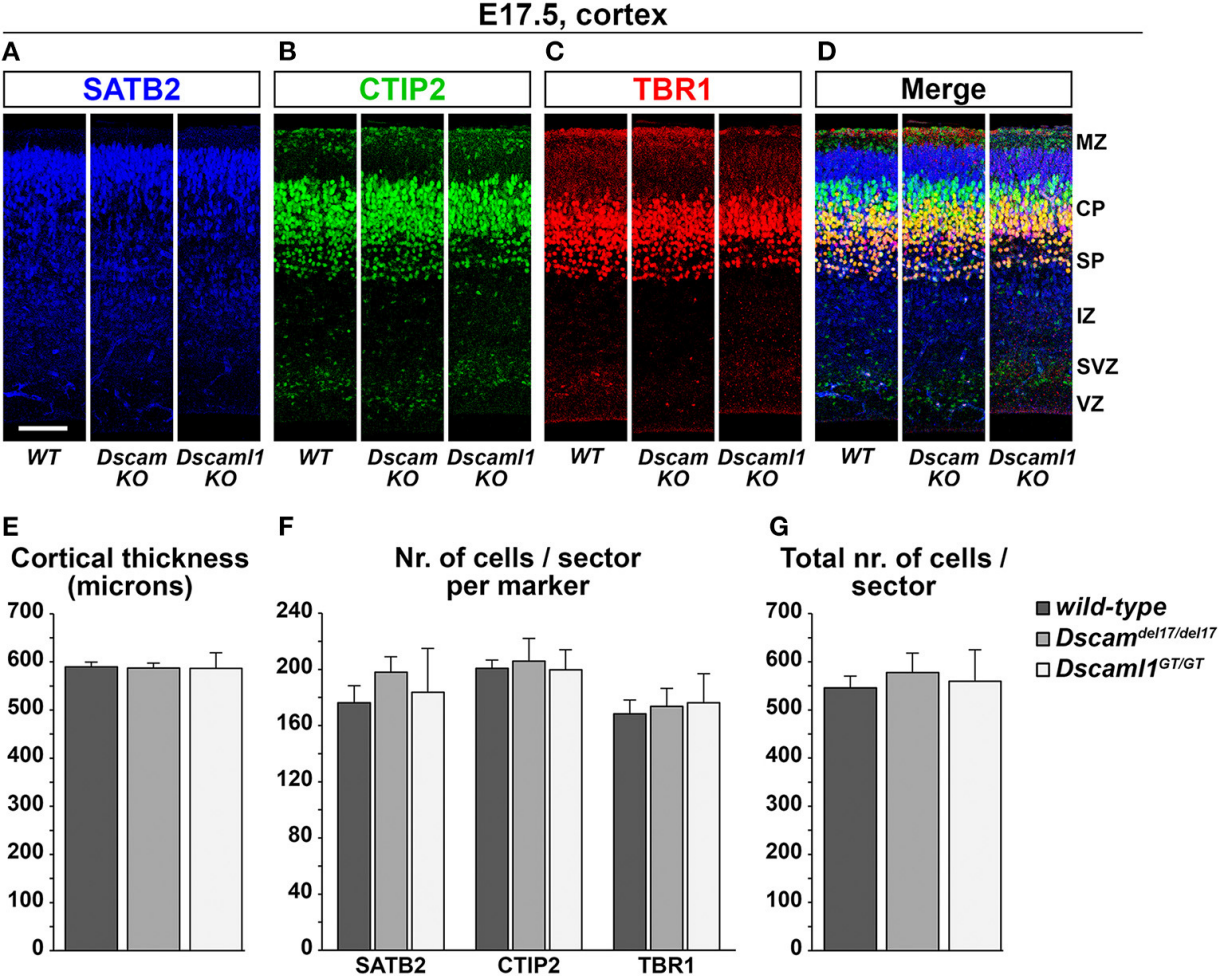
Normal cortical development and lamination in embryonic *Dscam* and *Dscaml1* null mutant mouse brains. **(A–D)** Triple immunostaining for the cortical markers SATB2 [upper layer neurons, blue; **(A)**], CTIP2 [deep layer neurons, green; **(B)**], and TBR1 [deep layer and subplate neurons, red; **(C)**] in coronal sections of wild-type (*WT*), *Dscaml1*^*del*17/*del*17^ (*Dscam KO*), and *Dscaml1*^*GT*/*GT*^ (*Dscaml1 KO*) E17.5 mouse brains. Panels represent radial sectors of the dorso-lateral cortex. **(E)** Histogram depicting average cortical thickness values measured across the rostro-caudal axis in wild-type, *Dscaml1*^*del*17/*del*17^, and *Dscaml1*^*GT*/*GT*^ E17.5 coronal mouse brain sections. No significant differences are detected across genotypes (*n* = 3 brains/group, mixed ANOVA test). **(F)** Histogram representing average numbers of SATB2+, CTIP2+, and TBR1+ cell measured in 100 μm-wide radial sectors of wild-type, *Dscaml1*^*del*17/*del*17^, and *Dscaml1*^*GT*/*GT*^ E17.5 coronal mouse brain sections across the rostro-caudal axis. No significant differences are detected across genotypes (*n* = 3 brains/group, mixed ANOVA test). **(G)** Histogram illustrating average total numbers of SATB2, CTIP2, and TBR1 immunolabeled cells measured in 100 μm radial sectors of wild-type, *Dscaml1*^*del*17/*del*17^, and *Dscaml1*^*GT*/*GT*^ E17.5 coronal mouse brain sections across the rostro-caudal axis. No significant differences are detected across genotypes (*n* = 3 brains/group, mixed ANOVA test). All graphs represent mean ± S.E.M values. CP, cortical plate; IZ: intermediate zone; MZ: marginal zone; SP: subplate; SVZ: subventricular zone; VZ: ventricular zone. Scale: **(A–D)**, 100 μm.

Expression data suggested that DSCAM and DSCAML1 are consistently present, albeit in different patterns, within the developing murine neocortex, and previous studies reported changes in cortical migration and thickness with *Dscam* or *Dscaml1* transcriptional suppression. These phenotypes were observed at early post-natal stages, implicating embryonic processes in the emergence of such defects. Thus, the development of cortical layers in *Dscam*^*del*17^ and *Dscaml1*^*GT*^ mutant mice was investigated at E17.5 *via* immunostaining with antibodies against the transcription factors Tbr1, Ctip2 (Bcl11b), and Satb2, markers, respectively of subplate and early born, early born, and late born cortical pyramidal neurons (Bulfone et al., 1995; Hevner et al., 2001; Leid et al., 2004; Arlotta et al., 2005; Britanova et al., 2008; Fishell and Hanashima, 2008) (Figures 5A–D). In parallel, cortical thickness was measured in all specimens to detect potential reductions in radial expansion. However, comparisons across *Dscam*^*del*1/*del*17^, *Dscaml1*^*GT*/*GT*^, and wild type mutant brains (*n* = 3 per genotype, three sections across the rostro-caudal axis per brain) did not unveil any significant differences in terms of total number of immunostained cells per 100-μm-wide tissue sector (Figure 5E) [*F*_genotype(2,6)_ = 0.126, *p* = 0.884], number of either Satb2+, Ctip2+, or Tbr1+ neurons per 100-μm-wide tissue sector (Figure 5F) [*F*_genotype(2,6)_ = 0.301, *p* = 0.750; *F*_genotype(2,6)_ = 0.070, *p* = 0.933; *F*_genotype(2,6)_ = 0.069, *p* = 0.934], and cortical thickness (Figure 5G) (*F*_genotype(2,6)_ = 0.008, *p* = 0.992). Moreover, statistical analysis of these measures at either anterior, intermediate, or posterior positions on the rostro-caudal axis yielded similar results, with a non-significant effect of genotype on cortical thickness (Supplementary Figure 3A) [*F*_position × *genotype*(4,12)_ = 0.300, *p* = 0.805], total number of immunostained cells *per sector* (not shown) [*F*_position × *genotype*(4,12)_ = 0.596, *p* = 0.673], and number of Satb2+, Ctip2+, or Tbr1+ cells *per sector* (Supplementary Figures 3B–D) (*F*_position × *genotype*(4,12)_ = 0.071, *p* = 0.990; *F*_position × *genotype*(4,12)_ = 1.226, *p* = 0.351; *F*_position × *genotype*(4,12)_ = 1.209, *p* = 0.357) at each rostro-caudal level. Taken together, these results point to a preserved overall development and lamination of the embryonic murine cortex in the absence of DSCAM or DSCAML1.

In summary, despite clear expression of DSCAM or DSCAML1 in cortical and subcortical areas, constitutive loss-of-function on the C57BL/6J background did not strongly affect cortical lamination, interneuron migration nor corticothalamic circuitry formation.

### *Dscam* or *Dscaml1* Gain of Function Affects the Embryonic Migration of Cortical Projection Neurons *in vivo*

Modeling DSCAM and DSCAML1 CNS overexpression in a mammalian species has the potential to unravel how disorders such as Down syndrome, in which DSCAM levels are known to be elevated in the fetal brain, and distal trisomy 11q, which involves the duplication of a chromosomal region including DSCAML1, develop in humans. Given the reduced cell number and anomalous neuronal organization observed within specific neocortex regions and layers of DS brains already at mid-to-late gestational stages (Colon, 1972; Becker et al., 1991; Golden and Hyman, 1994; Haydar and Reeves, 2012; Lott, 2012), we sought to examine the effects of *Dscam* and *Dscaml1* gain of function (GOF) within the developing mammalian neocortex. In particular, we focused on the process of radial migration of pyramidal neurons, since knockout/knockdown experiments in mouse brains pointed to possible roles of DSCAM and DSCAML1 in this context (Maynard and Stein, 2012; Zhang et al., 2015).

**Figure 6:**
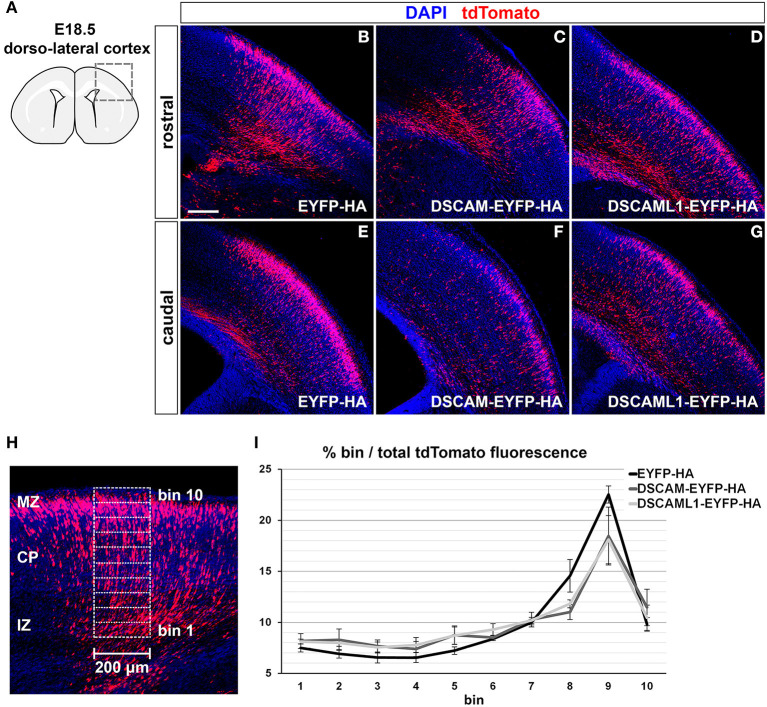
Effect of DSCAM and DSCAML1 *in vivo* gain of function in cortical projection neurons transfected with EYFP- and HA-tag labeled protein expression constructs. **(A–G)** Radial migration of cortical neuron electroporated with EYFP-HA (control) **(B,E)**, DSCAM-EYFP-HA **(C,F)**, or DSCAML1-EYFP-HA **(D,G)** expression constructs at E14.5. Electroporated cells are detected by co-expression of tdTomato in coronal sections of electroporated E18.5 mouse brains. Section are immunostained for tdTomato before analysis for signal enhancement. **(A)** Schematic representation of the dorso-lateral cortical region targeted via IUEP and represented in panels **(B–G)**, at two positions of the rostro-caudal axis. Radial distributions of tdTomato+ cells show mild differences between control and DSCAM/DSCAML1 expression construct-transfected specimens: tdTomato+ cells accumulate at the uppermost cortical layers in control conditions, whereas DSCAM and DSCAML1-overexpressing neurons spread more evenly across cortical zones. **(H,I)** Analysis of electroporated neurons' migration in E18.5 coronal brain sections. **(H)** tdTomato fluorescence intensity after immunostaining is measured in 200 μm-wide columns from the upper IZ to the MZ, subdivided radially in ten equally-sized bins. **(I)** Graph depicting average tdTomato fluorescence intensity levels per radial bin, expressed as percentages of total fluorescence intensity quantified over all ten bins, measured from brains electroporated with EYFP-HA (control), DSCAM-EYFP-HA, or DSCAML1-EYFP-HA expression constructs. A comparison of fluorescence distribution profiles highlights subtle, non-statistically significant differences in radial migration between control and tagged DSCAM/DSCAML1 expression conditions (*n* = 5–6 brains/group, mixed ANOVA test). Graph data represent mean ± S.E.M values. CP, cortical plate; IZ: intermediate zone; MZ: marginal zone. Scale: **(B–G)**, 200 μm.

GOF was thus modeled in the murine embryonic cortex *in vivo via in utero* electroporation (Tabata and Nakajima, 2001) of C-terminally-tagged DSCAM/DSCAML1 and tdTomato expression vectors (pCAGGS-DSCAM-EYFP-HA-IRES-tdTomato, pCAGGS-DSCAML1-EYFP-HA-IRES-tdTomato), while constructs driving the production of EYFP-HA and TdTomato were used in control specimens (pCAGGS-EYFP-HA-IRES-tdTomato). All constructs were pre-tested *in vitro* in mouse Neuro 2a cells, which were transfected and cultured for 2 days before imaging and protein extraction. Inspection of cellular resolution confocal images of successfully transfected (i.e., tdTomato-labeled) cells confirmed the synthesis and correct localization at cytoplasmic and membrane level of EYFP-tagged proteins (Supplementary Figures 4A–D); further investigation via Western Blot demonstrated the production of full length, EYFP- and HA-tagged DSCAM and DSCAML1 in the transfected Neuro 2a cells (Supplementary Figure 4E). *In utero* electroporation was performed at E14.5, leading to targeting of layer II/III and IV neurons (Takahashi et al., 1999; Taniguchi et al., 2012). Layer II/III neurons were reported to be the most affected around birth by DSCAM and DSCAML1 loss-of-function in the work of Maynard and Stein (2012) and Zhang et al. (2015), and layer II/III and IV neurons also show abnormalities in DS (Ross et al., 1984; Wisniewski and Rabe, 1986; Lott, 2012). Migration of tdTomato+ neurons was assessed in dorso-lateral cortical regions 4 days post-electroporation, at E18.5 (Figure 6A), once electroporated neurons had reached the uppermost regions of the CP in control conditions (Figures 6B,E). To verify the production and localization of the tagged DSCAM and DSCAML1 proteins in the transfected projection neurons, immunolabeling of EYFP in tdTomato+ neurons was examined in confocal images at cellular resolution. This analysis demonstrated the synthesis of EYFP-labeled proteins with both tagged DSCAM and DSCAML1 expression construct transfection, and an accumulation of these two molecules within cytoplasmic and plasma membrane compartments of the immature neurons' leading processes, particularly in soma-proximal areas (Supplementary Figures 4F,G).

Compared to EYFP-HA and tdTomato expressing neurons, which mostly accumulated in upper CP layers, cells expressing DSCAM-EYFP-HA or DSCAML1-EYFP-HA with tdTomato seemed to distribute across the cortical radial extension more sparsely (Figures 6B–G). To quantify this variation in distribution of tdTomato+ cells, tdTomato fluorescence was measured in columnar sectors, divided radially in 10 equal bins, of the electroporated cortices from the upper IZ to the MZ, and expressed for each bin as a percentage of total fluorescence (EYFP-HA group: *n* = 6; DSCAM-EYFP-HA group: *n* = 5; DSCAML1-EYFP-HA group: *n* = 5) (Figure 6H). While overall the tdTomato fluorescence distribution profiles across bins reflected the observed differences between control and DSCAM/DSCAML1 overexpression vector-transfected neurons (Figure 6I), statistical analysis indicated these profiles to non-significantly differ across electroporation groups over all bins (*F*_expression construct(2,13)_ = 0.518, *p* = 0.608; *F*_bin × *expression construct*(4.222, 27.443)_ = 1.423, *p* = 0.252). Our data suggest that DSCAML1 and DSCAM overexpression only has a mild effect on radial migration of projection neurons in the cortex during late embryonic development.

### *Dscam* or *Dscaml1* Gain of Function Impairs Migration and Process Development of Cortical Interneurons *in vitro*

**Figure 7:**
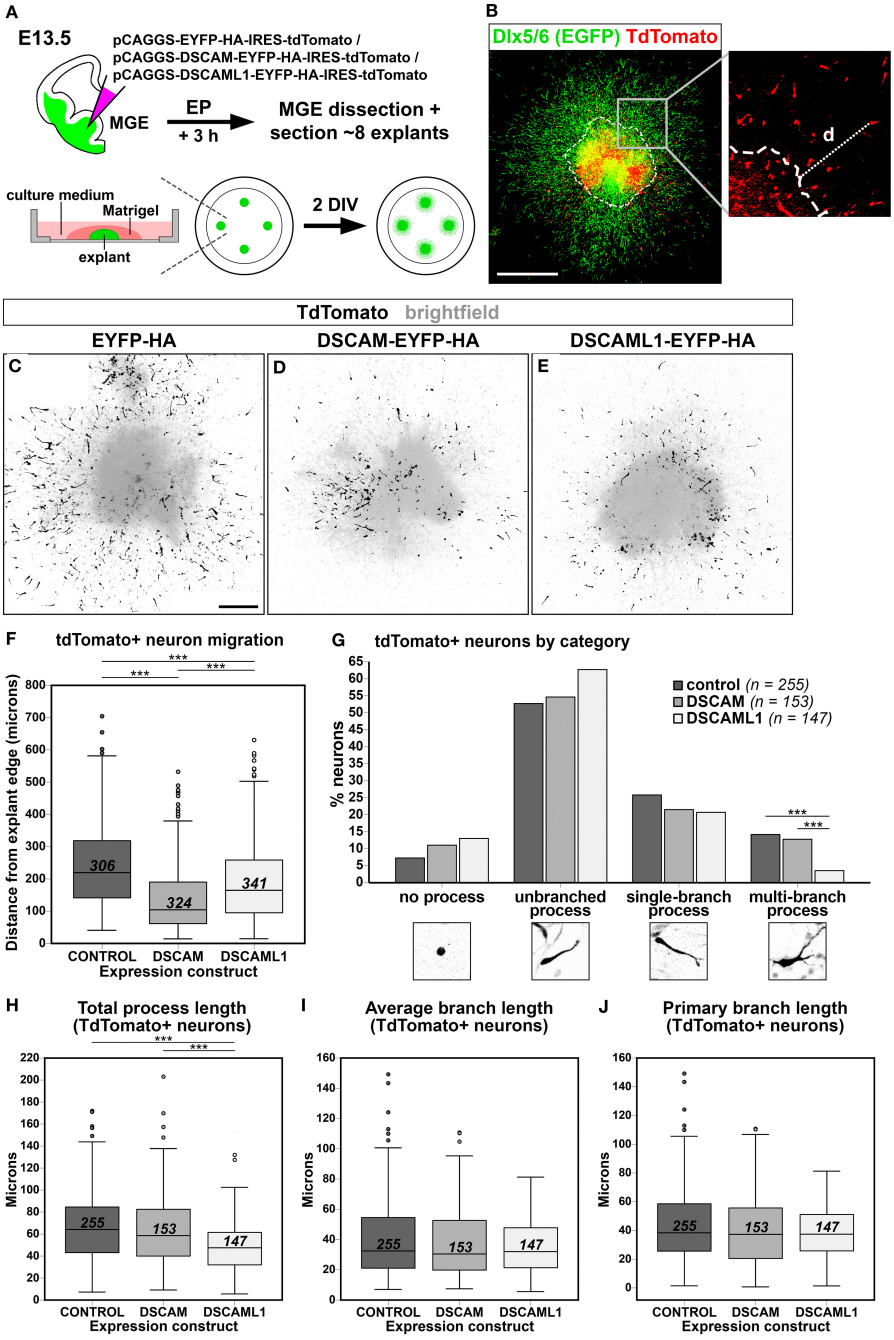
Effect of DSCAM and DSCAML1 *in vitro* gain of function in MGE-derived interneurons transfected with EYFP- and HA-tag labeled protein expression constructs. **(A)** Schematic illustration of the *ex vivo* MGE explant electroporation model used to study DSCAM and DSCAML1 overexpression effects on IN migration. E13.5 *Dlx5/6-Cre-IRES-EGFP* whole brains maintained in culture medium are injected at MGE level with expression constructs, electroporated, and dissected 3 h later to obtain MGE explants. The explants are embedded in Matrigel, supplemented with culture medium, and cultured for 48 h before analysis. **(B)** Analysis of IN migration in *Dlx5/6-Cre-IRES-EGFP* MGE explants 48 h post-electroporation with constructs driving co-expression of tagged proteins and tdTomato. The minimum distance from explant edge “d” is used as an indirect measure of migration capabilities of transfected (tdTomato+, red) INs, identifiable by EGFP expression (green). **(C–E)** Example images of cultured explants 48 h after electroporation of EYFP-HA (control) **(C)**, DSCAM-EYFP-HA **(D)**, or DSCAML1-EYFP-HA **(E)** expression constructs. **(F)** Boxplot chart depicting tdTomato+ neuron-associated “distance from explant edge” values measured in EYFP-HA (control), DSCAM-EYFP-HA (DSCAM), or DSCAML1-EYFP-HA (DSCAML1) expression constructs-transfected MGE explants. Comparisons across experimental groups reveal significantly shorter distances measured in either DSCAM or DSCAML1 expression construct-transfected vs. control construct-transfected explants, and in DSCAM vs. DSCAML1 expression construct-transfected explants (Kruskal-Wallis test with Dunn-Bonferroni pairwise comparisons, ***p < 0.001). **(G)** Histogram representing percentages of INs classified in one of four morphological categories, calculated on total number of INs analyzed per experimental group, in EYFP-HA (control), DSCAM-EYFP-HA (DSCAM), or DSCAML1-EYFP-HA (DSCAML1) expression constructs-transfected MGE explants. IN categories based on the type of leading process presented are illustrated on the x axis. Comparisons across experimental groups highlight a significantly lower percentage of cells possessing multi-branched processes in DSCAML1 expression construct-transfected vs. control or DSCAM expression construct-transfected explants (Pearson's Chi-square test and residuals analysis with Bonferroni correction; ***p < 0.001). **(H–J)** Boxplot chart illustrating tdTomato+ neuron-associated “total process length” **(H)**, “average branch length” **(I)**, and “primary branch length” **(J)** values measured in EYFP-HA (control), DSCAM-EYFP-HA (DSCAM), or DSCAML1-EYFP-HA (DSCAML1) expression constructs-transfected MGE explants. While no significant differences in average and primary branch length variables are detected across experimental groups, significantly shorter total process length are measured in DSCAML1 expression construct-transfected vs. either control or DSCAM expression construct-transfected explants (Kruskal-Wallis test with Dunn-Bonferroni pairwise comparisons, ***p < 0.001). In boxplot charts, numbers within boxes represent the total number of cells analyzed per experimental group; horizontal black lines within boxes denote median values; box edges indicate the 25th and 75th percentile of each group's distribution of values; whiskers represent highest and lowest values within 1.5 interquartile range measures per group; dots denote outliers. DIV, days *in vitro*. Scale: **(B)**, 500 μm; **(C–E)**, 200 μm.

In DS, defects in cortical layers II and III are associated to a striking reduction in the number of small granular cells, likely related to the GABAergic aspinous stellate cell type (Ross et al., 1984). Indeed, GABA neurotransmitter levels are decreased in DS brains at fetal developmental stages (Whittle et al., 2007). Moreover, in *Dlx1/2* double knockout mutant mice, in which immature INs migrating to the neocortex show an abnormal morphology in correspondence to an impaired tangential migration ability, embryonic subpallial *Dscam* mRNA levels are increased (Cobos et al., 2007). These findings suggest the possibility that overexpression of DSCAM proteins during embryonic development might impact the tangential migration or laminar positioning of cortical INs. We therefore modeled *Dscam* and *Dscaml1* GOF in mouse INs via *ex vivo* E13.5 MGE-targeted electroporation of C-terminally tagged DSCAM/DSCAML1 expression constructs (see section Dscam or Dscaml1 Gain of Function Affects the Embryonic Migration of Cortical Projection Neurons *in vivo*); subsequently, transfected IN migration was examined in Matrigel-embedded MGE explant cultures maintained *in vitro* for 2 days (Figure 7A). To easily identify MGE-derived INs, electroporation was performed in brains from *Dlx5/6-Cre-IRES-EGFP* mutant embryos, in which all postmitotic neurons express EGFP throughout embryonic development (Stenman et al., 2003). Migration was quantified by measuring distances traveled by transfected (i.e., tdTomato+) neurons from the edge of the explants (Figure 7B) in EYFP-HA (control; *n* = 306), DSCAM-EYFP-HA (DSCAM; *n* = 324), and DSCAML1-EYFP-HA (DSCAML1; *n* = 341) expression conditions. Example images of resulting explants are depicted in Figures 7C–E.

Statistical analysis indicated an overall difference in “distance from explant's edge” measures across treatment groups [*H*_(2)_ = 130.194, *P* < 0.001; mean distance rank scores: control = 612.49; DSCAM = 357,75; DSCAML1 = 488,96]. Pairwise *post-hoc* tests revealed that both overexpression of tagged DSCAM and DSCAML1 induced a significant reduction in IN spreading (*p* < 0.001 vs. control for both comparisons), with DSCAM overexpression leading to a more severe phenotype than DSCAML1 overexpression (*p* < 0.001) (Figure 7F).

Directed IN migration relies on the extension of a leading process (LP), which undergoes branching as the neuron explores the surrounding environment, paired with nucleokinesis; in turn, these processes are dependent on dynamic, extracellular cue-modulated microtubule and actin cytoskeleton remodeling events (Métin et al., 2006; Guo and Anton, 2014). Proper morphological development of the LP is thus essential for correct IN navigation, and interestingly several studies have provided a link between DSCAM-dependent intracellular signaling pathways or interactors and molecular networks controlling cytoskeletal remodeling (Liu et al., 2009; Purohit et al., 2012; Kamiyama et al., 2015; Okumura et al., 2015; Pérez-Núñez et al., 2016; Huo et al., 2018; Sachse et al., 2019). Thus, to gain insight into potential mechanisms underlying the migration defect observed in the explant EP assay previously described, we performed a morphological analysis of MGE-derived INs transfected with either control, tagged DSCAM, or tagged DSCAML1 expression constructs (*n* = 255, 153, 147). In particular, total LP length, average branch length, and primary branch length were measured for all neurons (Figures 7H–J), and each cell was categorized according to the morphology of the LP as “no process,” “unbranched process,” “single-branch process,” and “multi-branch process” (Figure 7G). This analysis overall revealed a significant effect of tagged DSCAML1 overexpression on IN morphology *ex vivo*. First, compared to control and DSCAM expression construct-transfected neurons, a significantly smaller percentage of INs transfected with DSCAML1 expression constructs represented “multi-branch process” cells [χ^2^_(6, N = 616)_ = 12.895757, *p* < 0.004]. Secondly, total LP length was found to be affected by the type of construct transfected [*H*_(2)_ = 40.467, *P* < 0.001]; *post-hoc* pairwise comparisons revealed this measure to be significantly reduced in DSCAML1 vs. control or DSCAM expression construct-transfected INs (*p* < 0.001), whereas no differences could be detected between DSCAM and control transfection groups (*p* = 0.380). However, no effect of expression construct transfected was observed on either average branch length [*H*_(2)_ = 1.436, *P* = 0.488] or primary branch length [*H*_(2)_ = 1.286, *P* = 0.526] measures.

In summary, while increased DSCAM levels significantly reduced IN spreading, we could not link this phenotype to obvious neurite growth or morphology defects. In contrast, we found the impaired migration resulting from DSCAML1 overexpression to be associated with a lower IN neurite branching complexity, suggesting different, yet related mechanisms of action of these molecules in INs.

## Discussion

This study first aimed to survey potential effects of generalized loss of DSCAM or DSCAML1 on cortical development, including lamination, patterning, and connectivity. The impact of such a loss seemed to be rather minimal. The absence of significant defects in forebrain patterning in DSCAM and DSCAML1 knockout mice was quite unexpected. Studying the cortical morphology of *Dscam*^*del*17^ mice, Maynard and Stein observed that homozygous mutant neonates exhibit an early post-natal (P1 to P10) reduction in cortical thickness attributable to a thinning of Cux1+ layers II and III, suggesting a specific role of DSCAM in the development of pyramidal neurons born around and after E14. This cortical phenotype was not accompanied by either an embryonic decrease in cell proliferation, tested via the administration of a BrdU pulse at E16.5, or an increased programmed cell death rate, assessed with a TUNEL assay at E16.5 and P1 (Maynard and Stein, 2012). As this reduced thickness was transient, it might have been caused by a delayed radial migration of the upper cortical layers. In a study where cortical expression of either *Dscam* or *Dscaml1* was suppressed *in vivo* via RNA interference, knockdown at E15.5 was found to impair radial migration of projection neurons at early post-natal stages, leading to a partial mispositioning of presumptive layer II/III neurons in layers IV/V observable for more than 2 weeks after birth (Zhang et al., 2015). Our data however could not substantiate a reduced thickness of upper cortical layers in the absence of DSCAM/DSCAML1, nor a significant defect in radial migration of late-born cortical neurons upon DSCAM/DSCAML1 overproduction, ruling out a major early role for DSCAMs in this process. The seeming discrepancy with the study of Maynard and Stein that detected reduced upper layer thickness at P1 might root in the different mouse background used, or in a specific defect in neurons populating the upper layers between E17.5 and P1. Whether DSCAMs might have a role in radial migration of a specific subpopulation of neurons, labeled by Cux1 but not Satb2 (Leone et al., 2015), needs further investigation. Considering the subtlety of the radial migration-related phenotypes examined, it is also possible that significant effects of a neuronal positioning delay/impairment due to *Dscam*/*Dscaml1* acute dosage variations embryonically might be detectable only at later developmental stages. Data relative to our IUEP-induced DSCAM/DSCAML1 overexpression experiments in particular suggest small, but evident changes in cortical migration dynamics being present 4 days after transfection with expression constructs. The high variability in results obtained via targeted electroporation, coupled with the limited time-frame within which additional DSCAM/DSCAML1 molecules were active, could have masked the impact of our genetic manipulations. Thus, it would be important to analyze the migration of transfected neurons during the first post-natal week in future research.

In the study by Zhang et al. (2015), acute downregulation of DSCAM or DSCAML1 during embryonic development also negatively affected callosal axonal outgrowth. Our findings indicate that loss of DSCAMs does not affect development of thalamocortical connectivity, suggesting that the negative impact of *Dscam* LOF on callosal connectivity might be a specific defect related to upper layer cortical neurons, rather than a general axonal outgrowth problem emerging in the absence of DSCAMs.

Absence of DSCAMs (particularly DSCAM) in vertebrates has been linked to either increased or decreased neurite outgrowth and branching, depending on the cellular context. Loss of DSCAM function in pyramidal cortical neurons drives a transient post-natal apical dendrite-associated branching and overall length increase, but a basal dendrite-associated branching and overall length decrease, *in vivo* (Maynard and Stein, 2012). Moreover, *Dscam* or *Dscaml1* knockdown results in an impaired axonal growth in cultured cortical neurons (Zhang et al., 2015). In retinal ganglion cells (RGCs), DSCAM downregulation leads to reduced axon extension and complexity levels in *Xenopus* (Santos et al., 2018), and delayed optic nerve outgrowth and thalamic targeting, accompanied by axonal fasciculation impairments, in mice (Bruce et al., 2017). Likewise, chick spinal cord interneurons present reduced axonal fasciculation levels upon DSCAM knockdown (Cohen et al., 2017). On the other hand, DSCAM knockout in *Xenopus* tectal neurons *in vivo* is associated with increased dendritic growth and branching rates. Interestingly, disruptive effects on axonal/dendritic growth and branching have been also observed upon DSCAM GOF. In mouse cortical neurons, *in vitro* overexpression of full-length DSCAM also impairs axonal outgrowth and branching dose-dependently, and increased expression of the DSCAM intracellular domain alone also results in a reduced overall neurite growth (Jain and Welshhans, 2016; Sachse et al., 2019). DSCAM overexpression additionally impairs dendritic branching and extension in mouse cultured hippocampal neurons (Alves-Sampaio et al., 2010) and in tectal neurons of *Xenopus* tadpoles (Santos et al., 2018). However, *Dscam* GOF is associated to RGC axonal overgrowth in mouse (Bruce et al., 2017). Overall, research on vertebrate development indicates that DSCAMs play important roles in outgrowth and branching of both dendrites and axons, and influence these processes in a markedly cell type-specific, and sometimes cellular structure-specific, manner. In our study, genetic manipulation of immature cortical inhibitory neurons *in vitro* resulted in a significant reduction of total LP length only upon DSCAML1 overexpression, an effect likely related to a concomitant impairment of LP branching. Nevertheless, both *Dscam* and *Dscaml1* GOF negatively impacted the migration process of post-mitotic INs away from progenitor cell territories in our explant model. The reduction of distances observed from neurons to explant edges with DSCAML1 overexpression could be directly due to the lower complexity and extension of the LP. Since this structure serves, similarly to the axonal growth cone, as an extracellular cue sensor that primarily orients the movement of migrating INs, defects in LP growth and branching can perturb the probing function of the LP, and lead to delayed and/or disorganized migration (Kappeler et al., 2006; Métin et al., 2006; Nasrallah et al., 2006; Martini et al., 2009; Valiente and Martini, 2009).

Interestingly, cortical INs derived from DS patient induced pluripotent stem cells (iPSC) have been reported to display smaller sizes, less complex neurite morphologies, and migration deficits *in vitro* as well as *in vivo* after transplantation in the mouse medial septum. Analysis of molecular pathways in these DS iPSC-derived GABAergic INS has revealed upregulation of PAK1, leading to increased phosphorylated cofilin levels. Pharmacological inhibition of this pathway restored DS iPSC-derived IN migration *in vitro*, suggesting a causal relation between PAK1 pathway dysregulation and migration defects (Huo et al., 2018). However, our explant EP results exclude significant morphological differences being determined by DSCAM overexpression in migrating INs. The different cell-autonomous effects observed upon DSCAM vs. DSCAML1 overexpression are consistent with the divergence of their intracellular domains, which are estimated to be only 45% identical at protein level and present unique interaction motifs (Agarwala et al., 2001; Fuerst et al., 2009; Cui et al., 2013; Pérez-Núñez et al., 2016). Indeed, the PAK1 binding domain located at the DSCAM C-terminal is one of the most divergent regions between DSCAM and DSCAML1 (Agarwala et al., 2001), thus it is unlikely that DSCAML1 would too activate this signaling pathway. Reduced distance of transfected INs from the explant core could derive from issues in cell-environment or cell-cell interactions. Imbalances in DSCAM-modulated signaling or adhesion might translate in an uncoordinated, non-linear IN migration away from the explant. Furthermore, *Dscam* GOF might affect other intracellular aspects of the migration process. Additional research is needed to investigate whether, for instance, IN nucleokinesis, saltatory motion dynamics, and centrosome positioning (Polleux et al., 2002; Bellion et al., 2005; Yanagida et al., 2012; Silva et al., 2018) are affected by dosage increases of both DSCAMs.

A general discrepancy in results between our experiments using constitutive LOF and transient, local overexpression models, as well as between our study and previous research, might point toward a mechanistic difference due to the nature of the models themselves, rather than the induced molecular dosage changes. Acute up- or downregulation via electroporation of specifically designed constructs creates a mosaic situation in which some cells have lost or gained DSCAM/DSCAML1, while untargeted cells in the local environment have not. Considering that DSCAMs interact homophilically, such a situation creates a transmembrane signaling protein-related imbalance across neurons that might exacerbate some phenotypes, as a complete LOF/GOF would not affect local cell-cell or cell-environment interaction dynamics, or might be compensated for by other membrane-bound molecules. In addition, electroporation of knockdown or overexpression constructs might induce undesirable toxicity effects on the targeted cells that need to be carefully controlled for.

Taken together, our findings suggest that DSCAM/DSCAML1 are rather dispensable in embryonic cortical development processes. Nevertheless, it is conceivable that dosage levels of a given cell might need to be in balance with those of neighboring cells to allow cell type-specific homophilic interactions. Future research will elucidate the molecular downstream effectors determining the subtle phenotypes observed in this study.

## Data Availability Statement

The original contributions presented in the study are included in the article/Supplementary Materials, further inquiries can be directed to the corresponding author/s.

## Ethics Statement

The animal study was reviewed and approved by Ethische Commissie Dierproeven, KU Leuven, project licenses 267/2015 and 005/2017.

## Author Contributions

ES, DS, and MM conceived the research project, and designed the experiments. MM performed all experiments together with AP, in case of MGE explant electroporation and culture assays. TA collected samples and helped in the revision. MM analyzed experimental results and data. SS produced plasmid constructs for the subcloning of *Dscam-EYFP-HA* and *Dscaml1-EYFP-HA* sequences. RV and LN provided technical support in the performance of ISH and IHC experiments, and carried out genotyping of all tissue samples. MM and ES wrote the manuscript in consultation with DS. All authors contributed to the article and approved the submitted version.

## Conflict of Interest

The authors declare that the research was conducted in the absence of any commercial or financial relationships that could be construed as a potential conflict of interest.
