# Supplementary material for: Subtle Roles of Down Syndrome Cell Adhesion Molecules in Embryonic Forebrain Development and Neuronal Migration
Source: Front Cell Dev Biol. 2021 Jan 28;8:624181. doi: 10.3389/fcell.2020.624181 (PMC7876293; doi:10.3389/fcell.2020.624181)
Supplement: Supplementary file 1 [file Data_Sheet_1.pdf]

Supplementary Material

Table S1 – Genotyping primers and relative PCR product sizes

Target gene	Primers	PCR products
Dscam	Forward:<br>5'- CTTTGCGCGTTATGATCCT-3'<br><br>Reverse:<br>5'- GTGGTGTCGATACTGATG-3'	Wild-type allele:<br>170 bp<br><br>*Dscam*^{del17} allele:<br>132 bp
Dscaml1	Forward:<br>5'- ATGCCACTGTGCCTGGCTGTT-3'<br><br>Reverse (wild-type allele):<br>5'- CCCAGCAGTTGAGTGCCCTGG-3'<br>Reverse (*Dscaml1*^{GT} allele):<br>5'-TATCCACAACCAACGCACCCAAGC-3'	wild-type allele:<br>0.4 Kb<br><br>*Dscaml1*^{GT} allele:<br>0.3 Kb

Table S2 – NEPA21-specific electroporation parameters used for IUEP

Pulse type	Voltage (V)	Length (ms)	Interval (ms)	Number of pulses	Decay rate (%)	Polarity reversal
Poring	37	30	450	1	10	off
Transfer	34	50	450	6	40	off

Table S3 – BTX-specific electroporation parameters used for MEP

Voltage (V)	Length (ms)	Interval (ms)	Number of pulses	Polarity reversal
50	50	1000	5	off

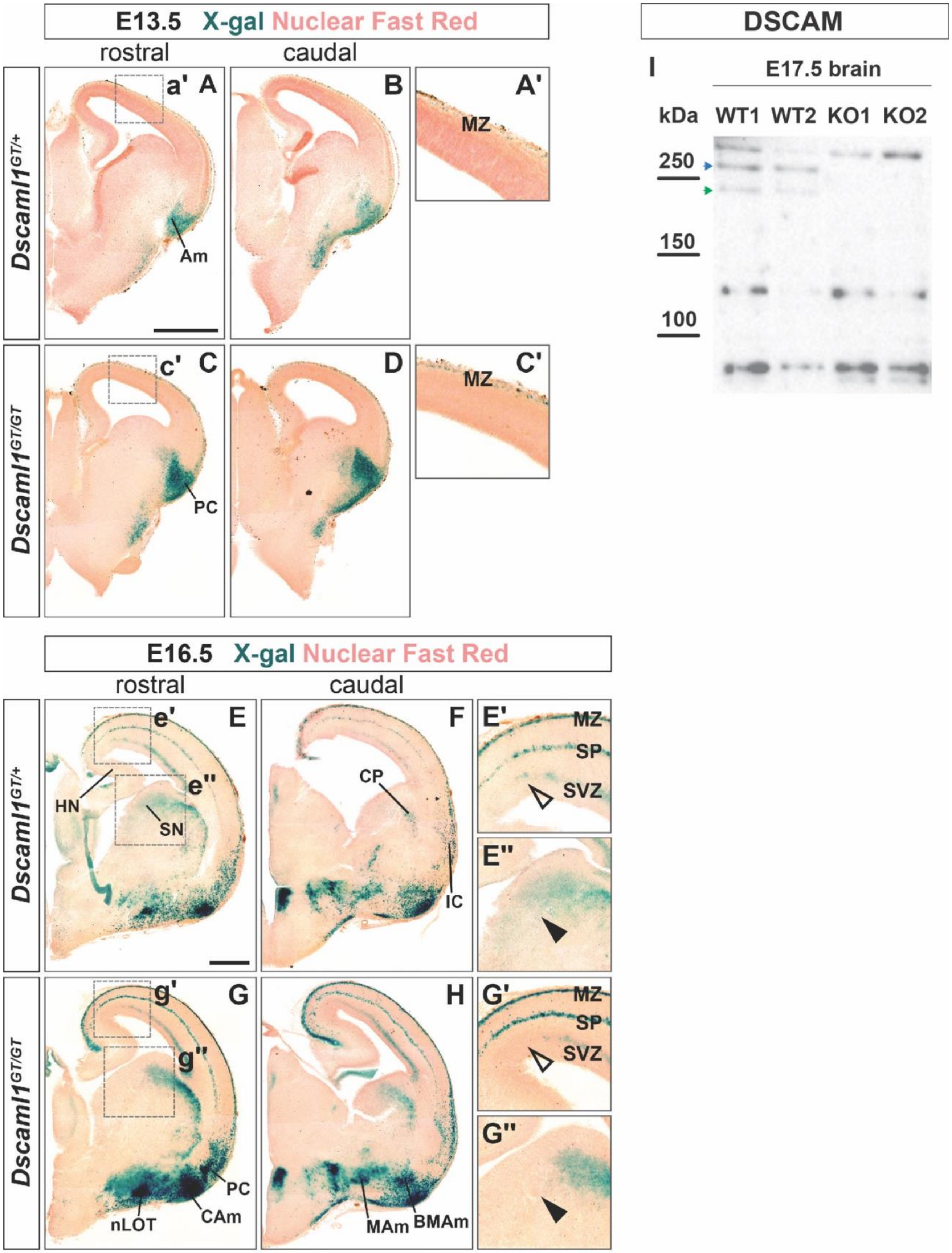
E13.5 X-gal Nuclear Fast Red
rostral
caudal
Dscaml1GT/+
Dscaml1GT/GT
a' A
B
A'
MZ
Am
c' C
D
C'
MZ
PC
DSCAM
I
E17.5 brain
kDa
WT1 WT2 KO1 KO2
250
150
100
E16.5 X-gal Nuclear Fast Red
rostral
caudal
e' E
e''
HN
SN
F
CP
IC
E'
MZ
SP
SVZ
E''
g' G
g''
PC
nLOT
CAm
H
MAm
BMAm
G'
MZ
SP
SVZ
G''

Supplementary Figure 1 – Synthesis of DSCAML1 detected via X-gal staining in the embryonic *Dscaml1^{GT}* mouse forebrain.

(A–D) X-gal staining patterns, identifying cell populations producing DSCAML1–β-galactosidase non-functional fusion protein, in coronal sections of *Dscaml1$^{GT/+}$* and *Dscaml1$^{GT/GT}$* E13.5 mouse brains. DSCAML1 is limitedly synthetized in post-mitotic regions of the *Dscaml1$^{GT/+}$* E13.5 telencephalon across the rostro-caudal axis. In subpallial regions, X-gal staining, associated to the production of non-functional DSCAML1–β-galactosidase fusion proteins, is detected mostly in cell populations at the pial surface; sparse staining is also found within the presumptive striatum. Maximal staining intensities are observed in presumptive amygdala territories, and in the developing piriform cortex (A, B). In the *Dscaml1$^{GT/+}$* E13.5 dorso-lateral cortex, X-gal staining can be exclusively detected in the MZ (A'). Similar patterns are observed in *Dscaml1$^{GT/GT}$* brains, both at subpallial / ventral cortical (C, D) and dorso-lateral cortical (C') level.

(E–G) X-gal staining patterns in coronal sections of *Dscaml1$^{GT/+}$* and *Dscaml1$^{GT/GT}$* E16.5 mouse brains. DSCAML1 production occurs both in post-mitotic areas and in subventricular progenitor zones of the *Dscaml1$^{GT/+}$* E16.5 telencephalon. Within the vTel, sparse X-gal staining can be observed in a transition area between the SVZ and the mantle zone, corresponding to striatal neuroepithelium cells, and in the lateral caudate-putamen. High, dense staining can be furthermore detected in medial, basomedial, and cortical nuclei of the developing amygdala, surrounded by sparser staining, as well as in the nucleus of the lateral olfactory tract, and in piriform cortical areas (E, F). In the *Dscaml1$^{GT/+}$* E16.5 cortex, X-gal staining delineates three layers, approximately corresponding to the MP, the SP, and the SVZ, which extend tangentially to reach hippocampal neuroepitelium regions (E'). Staining patterns in *Dscaml1$^{GT/GT}$* sections overall match those observed in *Dscaml1$^{GT/+}$* specimens (G, H). However, in the developing cortex, staining within the SVZ appears to be missing in a restricted area most proximal to the hippocampal neuroepithelium in *Dscaml1$^{GT/GT}$* versus *Dscaml1$^{GT/+}$* sections (E' and G', empty arrowheads). Furthermore, diffuse subpallial staining in the *Dscaml1$^{GT/GT}$* SVZ/mantle transition region, in presumptive striatal territories, does not expand medially as seen in *Dscaml1$^{GT/+}$* samples (E'' and G'', filled arrowheads).

(I) Western blot of E17.5 brain lysates of wild type (WT) and *Dscam$^{del17/del17}$* (KO) brains reveals the absence of bands at 220kDa (predicted molecular weight of DSCAM) in the KO samples (green arrow). A second band of larger molecular weight is also missing in the KO and might represent a post-translationally modified form of DSCAM (blue arrow).

Am, amygdala; BMAm, basomedial nucleus of the amygdala; CAm, cortical nucleus of the amygdala; CP, caudate-putamen; HN, hippocampal neuroepithelium; IC, insular cortex; MAm, medial nucleus of the amygdala; MZ, marginal zone; nLOT, nucleus of the lateral olfactory tract; PC, piriform cortex; SN, striatal neuroepithelium; SP, subplate; SVZ, subventricular zone; VZ, ventricular zone.

Scale: A–D, 500 μm; E–H, 500 μm.

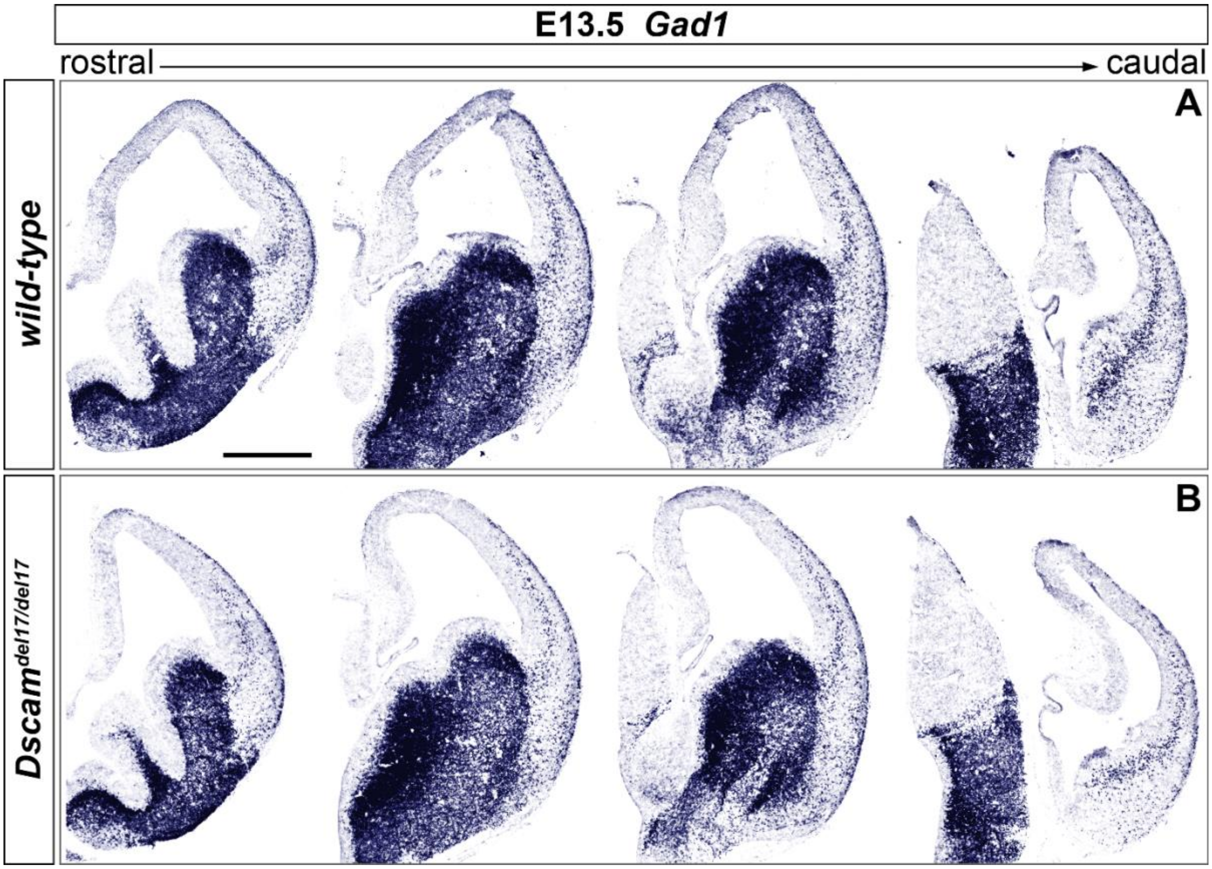

Supplementary Figure 2. Interneuron migration in *Dscam* null mutant mouse brains.
(A–B) *Gad1* mRNA expression, detected by ISH with antisense RNA probes, in coronal sections of wild-type (A) and *Dscam*$^{del17/del17}$ E13.5 mouse brains. In both cases, *Gad1*-expressing cells are found in the ventral telencephalon, as well as in two streams of cortical interneurons that migrate tangentially into the cortical field.
Scale: A–B, 500 µm.

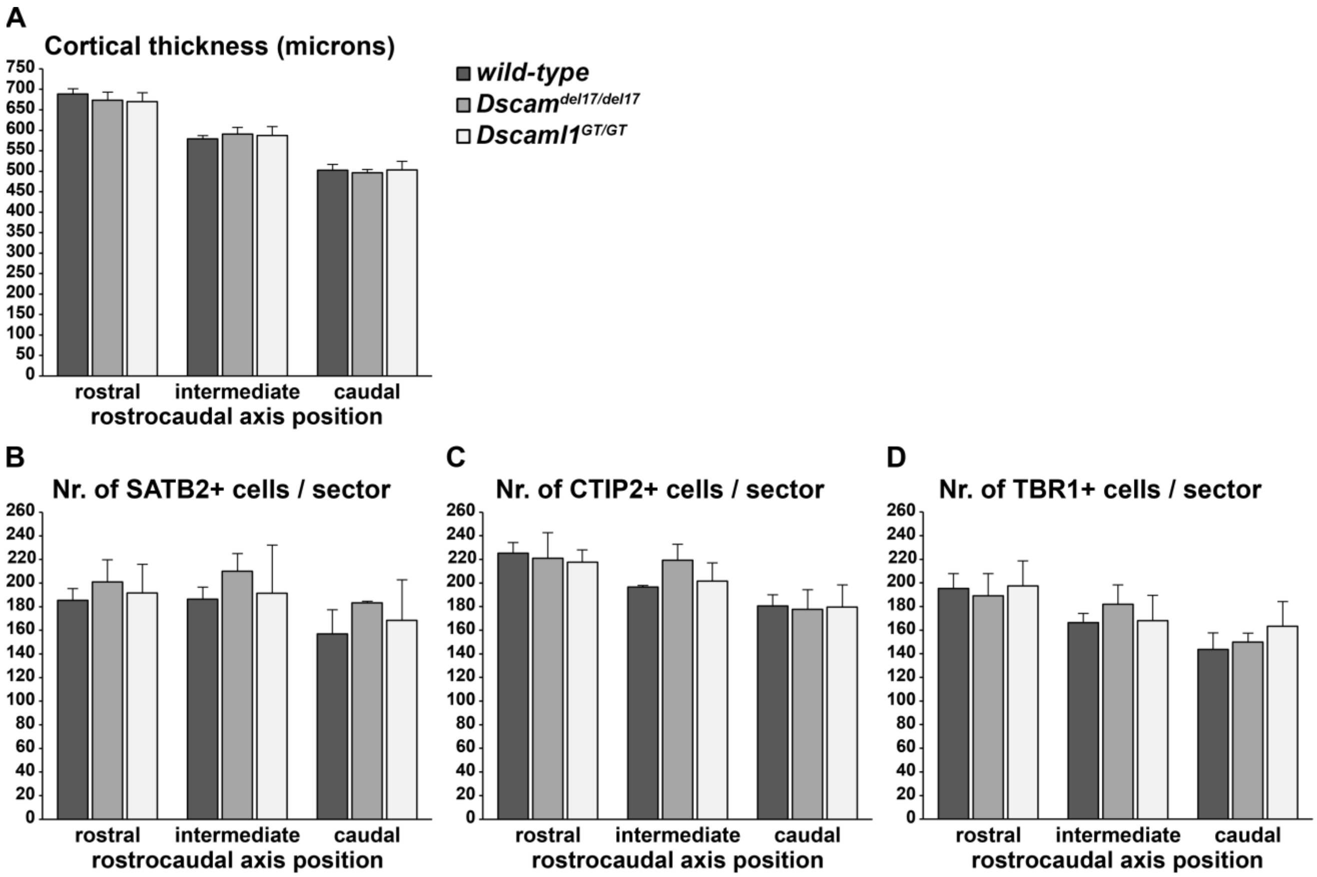

Supplementary Figure 3 – Normal cortical development and lamination across the rostro-caudal axis in embryonic *Dscam* and *Dscaml1* null mutant mouse brains.
(E) Histogram depicting average cortical thickness values separately measured in three rostro-caudal axis positions (rostral, intermediate, caudal) in wild-type, *Dscaml1*$^{del17/del17}$, and *Dscaml1*$^{GT/GT}$ E17.5 coronal mouse brain sections. No significant differences are detected across genotypes (*n* = 3 brains/group, mixed ANOVA test).
(F) Histogram representing average numbers of SATB2+, CTIP2+, and TBR1+ cell measured in 100 µm-wide radial sectors of wild-type, *Dscaml1*$^{del17/del17}$, and *Dscaml1*$^{GT/GT}$ E17.5 coronal mouse brain sections representative of three rostro-caudal axis positions (rostral, intermediate, caudal). No significant differences are detected across genotypes (*n* = 3 brains/group, mixed ANOVA test).
(G) Histogram illustrating average total numbers of SATB2, CTIP2, and TBR1 immunolabeled cells measured in 100 µm radial sectors of wild-type, *Dscaml1*$^{del17/del17}$, and *Dscaml1*$^{GT/GT}$ E17.5 coronal mouse brain sections representative of three rostro-caudal axis positions (rostral, intermediate, caudal). No significant differences are detected across genotypes (*n* = 3 brains/group, mixed ANOVA test).
All graphs represent mean ± S.E.M values.

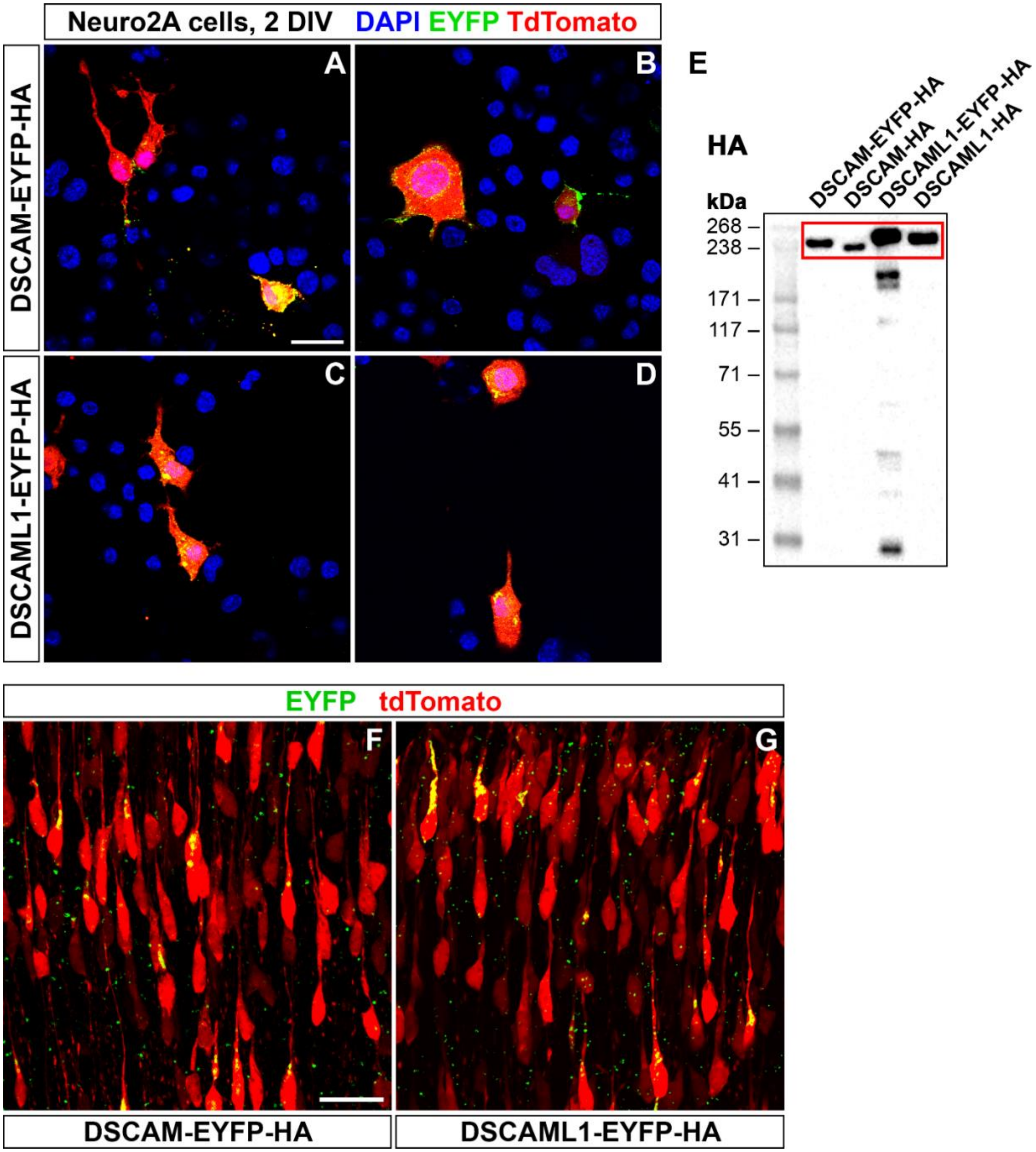

Supplementary Figure 4. – *In vitro* and *in vivo* expression and cellular localization of tagged DSCAM and DSCAML1 post-transfection with pCAGGS expression constructs.

(A–B) Neuro 2a cells cultured for 48 h after transfection with DSCAM-EYFP-HA (A, B) or DSCAML1-EYFP-HA (C, D) expression constructs. EYFP fluorescence (green), corresponding to tagged DSCAM and DSCAML1 molecules, can be observed mostly in the cytoplasm and plasma membranes of efficiently transfected cells, indicated by tdTomato fluorescence (red); in differentiating cells, EYFP-labeled proteins can be detected in immature neurites (B, C).

(E) Western blot using anti-HA-tag antibodies on protein fractions from Neuro 2a cells transfected with either DSCAM-EYFP-HA, DSCAM-HA, DSCAML1-EYFP-HA, DSCAM-HA expression

constructs. In all samples analyzed, protein bands in the correct size of around 250, 220, 255, and 235 KDa, respectively, were detected (red box).

(F–H) Cellular resolution magnifications of CP areas obtained from coronal sections of E18.5 brains electroporated with DSCAM-EYFP-HA (F) or DSCAML1-EYFP-HA (G) expression constructs at E14.5, and immunostained for EYFP and tdTomato. Production of EYFP-labeled DSCAM and DSCAML1 (green) can be clearly observed in transfected cortical neurons, identified by whole-cell tdTomato labeling (red); labeled molecules localize mostly at cytoplasmic and plasma membrane level, and accumulate along the leading processes of migrating projection neurons, especially in soma-proximal areas.

DIV, days in vitro.

Scale: A–D, 30 μm; F–G, 25 μm
